# The 5-HT_2C_ receptor agonist meta-chlorophenylpiperazine (mCPP) reduces palatable food consumption and BOLD fMRI responses to food images in healthy female volunteers

**DOI:** 10.1007/s00213-017-4764-9

**Published:** 2017-10-28

**Authors:** Jason M. Thomas, Colin T. Dourish, Jeremy Tomlinson, Zaki Hassan-Smith, Peter C. Hansen, Suzanne Higgs

**Affiliations:** 1 School of Psychology, University of Birmingham, Edgbaston, Birmingham, B15 2TT UK; 2 Department of Psychology, Aston University, Birmingham, B4 7ET UK; 3 P1vital, Manor House, Howbery Park, Wallingford, Oxfordshire, OX10 8BA UK; 4 Oxford Centre for Diabetes, Endocrinology and Metabolism, Oxford NIHR Biomedical Research Centre, Churchill Hospital, University of Oxford, Headington, OX7 3LJ UK; 5 Centre for Endocrinology, School of Clinical and Experimental Medicine, University of Birmingham, Birmingham, B15 2TH UK

**Keywords:** 5-HT_2C_, Food consumption, BOLD fMRI

## Abstract

**Rationale:**

Brain 5-HT_2C_ receptors form part of a neural network that controls eating behaviour. 5-HT_2C_ receptor agonists decrease food intake by activating proopiomelanocortin (POMC) neurons in the arcuate nucleus of the hypothalamus, but recent research in rodents has suggested that 5-HT_2C_ receptor agonists may also act via dopaminergic circuitry to reduce the rewarding value of food and other reinforcers. No mechanistic studies on the effects of 5-HT_2C_ agonists on food intake in humans have been conducted to date.

**Objectives:**

The present study examined the effects of the 5-HT_2C_ receptor agonist meta-chlorophenylpiperazine (mCPP) on food consumption, eating microstructure and blood oxygen level-dependent (BOLD) functional magnetic resonance imaging (fMRI) responses to food pictures in healthy female volunteers.

**Methods:**

In a double-blind, placebo-controlled, crossover design, participants were randomized immediately after screening to receive oral mCPP (30mg) in a single morning dose, or placebo, in a counterbalanced order. Test foods were served from a Universal Eating Monitor (UEM) that measured eating rate and fMRI BOLD signals to the sight of food and non-food images were recorded.

**Results:**

mCPP decreased rated appetite and intake of a palatable snack eaten in the absence of hunger but had no significant effect on the consumption of a pasta lunch (although pasta eating rate was reduced). mCPP also decreased BOLD fMRI responses to the sight of food pictures in areas of reward-associated circuitry. A post hoc analysis identified individual variability in the response to mCPP (exploratory responder-non-responder analysis). Some participants did not reduce their cookie intake after treatment with mCPP and this lack of response was associated with enhanced ratings of cookie pleasantness and enhanced baseline BOLD responses to food images in key reward and appetite circuitry.

**Conclusions:**

These results suggest that 5-HT_2C_ receptor activation in humans inhibits food reward-related responding and that further investigation of stratification of responding to mCPP and other 5-HT_2C_ receptor agonists is warranted.

**Electronic supplementary material:**

The online version of this article (10.1007/s00213-017-4764-9) contains supplementary material, which is available to authorized users.

## Introduction

Recent work on the neurophysiological basis of eating behaviour suggests that there are close interactions between the homeostatic networks that respond to changes in metabolic state and those involved in assigning reward value to motivational stimuli and translating motivation into action (Berthoud 2011). For example, food deprivation increases the incentive value of food, which is reflected in enhanced responses to appetitive stimuli in reward-related brain areas whereas satiation decreases responses in reward-related circuitry (Goldstone et al. 2009; Thomas et al. 2015; see van der Laan et al. 2011 for a meta-analysis). These effects are likely to be mediated by the action of metabolic signals such as leptin, insulin, peptide YY (PYY) and ghrelin on the activity of the mesocorticolimbic dopamine system (Batterham et al. 2007; Guthoff et al. 2010; Farooqi et al. 2007; Malik et al. 2008).

The role of serotonin in the control of appetite has been largely interpreted within a framework of homeostatic eating, and the influence of hypothalamic cellular mechanisms in the effects of serotonergic drugs on food intake is well documented. The melanocortin system of the arcuate nucleus of the hypothalamus (ARC) has been identified as a key network in the anorectic effects of serotonin agonists, including the 5-HT_2C_ receptor agonist lorcaserin, which has recently been approved by the FDA to treat obesity (Heisler et al. 2002, 2006; Sohn et al. 2011). However, alterations in serotonin transmission also affect reward-related circuits in the brain to influence food intake, either directly (Aronson et al. 1995) or indirectly via modulation of dopamine activity. Indeed, 5-HT_2C_ receptors expressed in the ventral tegmental area (VTA) (Bubar and Cunningham 2007) modulate activity of dopaminergic (DA) projections to the nucleus accumbens (NAcc) to alter motivation for food and drug reinforcers in rats (Fletcher et al. 2004; Higgins et al. 2013). These preclinical data suggest a specific role for 5-HT_2C_ receptor activation in linking hypothalamic energy-sensing mechanisms to motivational aspects of eating behaviour. However, to date, no mechanistic studies on the effects of 5-HT_2C_ receptor agonists on food intake in humans have been reported.

Our aim here is to test the hypothesis that 5-HT_2C_ receptor activation reduces food-reward responses in humans. We investigate for the first time the effect of the preferential 5-HT_2C_ receptor agonist meta-chlorophenylpiperazine (mCPP) on hedonic eating and neural responses to food images using functional magnetic resonance imaging (fMRI). mCPP is known to reduce appetite in humans (Cowen et al. 1995; Walsh et al. 1994; Thomas et al. 2014) but to probe reward-related effects of mCPP, we examine the effect of mCPP on the consumption of a staple meal consumed in a hungry state and a palatable high-energy dense snack food eaten in the absence of hunger. We also examine the microstructure of eating using a universal eating monitor (UEM) to identify specific behavioural changes that may underlie decreases in food intake (Thomas et al. 2014). We predicted that mCPP would reduce rated appetite (Thomas et al. 2014), but that the drug effect on intake would be greater for the palatable food consumed in the absence of hunger than for the staple food consumed when hungry. We also predicted that this effect of mCPP on palatable food consumption might be mediated by a reduction in rated pleasantness of the food and reflected in a reduction of markers of neural activation in reward-related brain circuitry.

## Materials and methods

### Participants

Twenty-four women volunteers were recruited from the University of Birmingham. Posters advertised the study as an “Appetite & fMRI study”, and participants were compensated with cash or course credits upon completion. The sample size was based on the results of a previous study (Thomas et al. 2014). Ethical approval was provided by South Birmingham Research Ethics Committee (National Research Ethics Service number 11/WM/0411) and informed consent was provided by all participants. Participants were screened to exclude the following: under 18 or over 65 years old, body mass index (BMI) under 18.5 or over 24.9 kg/m^2^, English not the first language, taking any psychotropic medication or recreational drugs, past or current Axis 1 disorder (determined by the Structured Clinical Interview for DSM-IV Axis I Disorders; SCID-I/P; Spitzer et al. 2004), pregnant or breastfeeding, smoker, dyslexic, food allergies, diabetic, cognitive dietary restraint score higher than 10 as measured by the Three-Factor Eating Questionnaire (TFEQ; Stunkard and Messick 1985). Participants were also excluded if they had previously taken part in a mCPP study, were left-handed or had any contraindications to fMRI scanning. Women were asked to participate in test days that fell outside their premenstrual week.

### Design

In a double-blind, placebo-controlled, crossover design, participants were randomized immediately after the screening days to receive oral mCPP (30 mg) (Thomas et al. 2014) in a single morning dose, or placebo, in a counterbalanced order. mCPP and the matched placebo were supplied by the Guy’s and St Thomas’ NHS Foundation Trust Pharmacy Manufacturing Unit. The washout period between test sessions was 7 days. To maintain blinding, mCPP and placebo were prepared in identical capsules and unblinding occurred on study completion. Peak plasma levels of mCPP are observed 120–180 min after oral administration, which was timed to coincide with the second fMRI scan.

### Universal eating monitor

Food was served on a UEM consisting of a balance (Sartorius Model CP4201, Sartorius Ltd., Epsom, UK; 0.1 g accuracy) placed underneath the surface of a table and connected to a laptop computer. A placemat hid the balance from view (Thomas et al. 2014).

#### Pasta

Dishes filled with 220 g of pasta were provided. Each time the participant ate 50 g of pasta, the Sussex Ingestion Pattern Monitor (SIPM) software (version 2.0.13) interrupted the participant to complete computerised visual analogue scale (VAS) ratings (hunger, fullness and pleasantness of the pasta). After consuming 150 g, participants were interrupted and provided with a fresh dish of 220 g of pasta. Participants were asked to eat in this manner until they felt ‘comfortably full’. The lunch consisted of pasta shells in a tomato and herb sauce served at 55–60 °C (207 kcal per 220-g serving).

#### Cookies

Bowls containing 80 g of cookie pieces were provided. Each time the participant ate 10 g of cookie pieces, the SIPM software interrupted the participant to complete VAS ratings as described above. After consuming 60 g, participants were interrupted and provided with a fresh bowl containing 80 g of cookie pieces. Participants were asked to eat until they felt ‘comfortably full’. The cookies were Maryland Chocolate Chip Cookies, with each cookie being broken into 6–7 pieces (390 kcal per 80-g serving).

### Salivary cortisol assessment

Salivary cortisol was collected to confirm a pharmacological response to mCPP administration (Meltzer and Maes 1995) and was measured by liquid chromatography–mass spectrometry (LC–MS/MS) as described previously (Thomas et al. 2014).

### Procedure

**Fig. 1:**
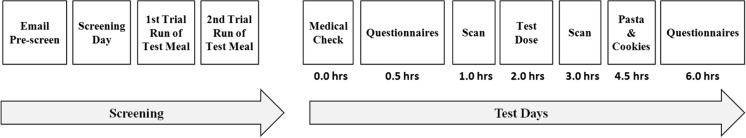
Flow diagram for screening process followed by an overview of key events and timings for test days in hours (hrs)

The experimental procedure is summarised in Fig. 1.

#### Screening days

Participants who met the study criteria were invited to a screening day at which they completed: a medical screening sheet, the Eysenck Personality Questionnaire (EPQ; Eysenck and Eysenck 1975) and a questionnaire to determine whether they usually consume lunch. Height and weight were also taken to calculate BMI. Participants returned after a week for two practice sessions, both a week apart, with the UEM.

#### Test days

Participants arrived having consumed their normal breakfast. If they passed a medical examination (first day only), they were breathalysed and completed a pregnancy test, before completing the first batch of questionnaires to assess what and when they had eaten that morning, and several measures to assess mood: Beck Depression Inventory (BDI; Beck et al. 1961); Befindlichskeit Scale of mood and energy (BFS; von Zerssen et al. 1974); Positive and Negative Affect Schedule (PANAS; Watson et al. 1988); and State-Trait Anxiety Inventory (STAI; Spielberger 1983). They also completed the Power of Food Scale (PFS, Lowe et al. 2009), and the Barratt Impulsivity Scale (BIS—Patton et al. 1995) and baseline VAS to assess the following: ‘alertness’; ‘disgust’; ‘drowsiness’; ‘light-headed’; ‘anxiety’; ‘happiness’; ‘nausea’; ‘sadness’; ‘withdrawn’; ‘faint’; ‘hungry’; ‘full’; ‘desire to eat’; and ‘thirst’.

A baseline fMRI scan was then conducted, after which participants completed the VAS, provided a saliva sample and took either mCPP or placebo. At 30 min post-dosing, they completed another set of VAS. Thirty minutes later, participants provided a saliva sample and completed VAS. They were scanned again and then completed a set of VAS and provided another saliva sample.

Immediately before lunch, participants completed VAS and were given ad libitum access to a pasta lunch via the UEM. After lunch, participants completed VAS followed by a 20-min break, after which a further set of VAS was completed before participants were given ad libitum access to a cookie snack. Immediately after the snack, participants filled out VAS. Approximately 30–40 min later, participants completed a second batch of questionnaires: VAS, BDI, BFS, PANAS, STAI, PFS and BIS and provided a final saliva sample, rated the scanner task food images and had a single blood sample taken. At the end of their second session, participants were fully debriefed, thanked for their time and reimbursed for participation.

### Imaging task

The scanner task was based on that used by Allen et al. 2016. There were three separate blocks in which participants viewed 40 food pictures (20 high-calorie food images and 20 low-calorie food images), 40 non-food control pictures and 5 smiley face images in three separate blocks. The high-calorie images (mean number of calories per 100 g of food shown = 365) and low-calorie images (mean number of calories per 100 g of food shown = 79) were comparable with previously published tasks (Goldstone et al. 2009). The pictures were displayed for 2500 ms, fixation points were displayed for 3500 ms and smiley face images were displayed for 1500 ms. Participants were asked to pay attention to all images, but to imagine eating the foods they saw during the task, and to press a button on a button box when they saw a smiley face to ensure they were maintaining attention.

### fMRI data acquisition and analysis

An event-related design was used, in which the stimuli were presented in pseudo random sequence. The scanner was a 3.0 T Achieva (Philips) whole body scanner with an eight-channel head coil. T2*-weighted echo planar imaging (EPI) slices were acquired every 2.5 s (TR = 2.5). Thirty-three axial slices with an in-plane resolution of 2.5 × 2.5 × 3 mm^2^ and slice thickness of 3 mm (no gap) were acquired, with a matrix size of 96 × 96 and field of view of 240 × 240 mm. Acquisition angulation was consistently AC-PC. Two hundred volumes were acquired for each block of the task with two dummy scans which were discarded prior to any analysis. A whole brain T2*-weighted EPI volume (resting state) was also acquired (AC-PC angulation), along with an anatomic T1-weighted volume acquired in the sagittal plane slice thickness of 1 mm and in-plane with a reconstructed resolution of 1.0 × 1.0 × 1.0 mm. The FMRIB software library (FSL; FMRIB, Oxford, www.fmrib.ox.ac.uk/fsl) was used for pre-processing and data analyses. Pre-processing involved high-pass filter cutoff of 60 s; motion correction using FMRIB’s Linear Image Registration Tool (MCFLIRT); motion parameters as regressors of no interest; interleaved slice timing correction; spatial smoothing with a 6-mm full-width-half-maximum kernel; high-pass temporal filtering and FILM pre-whitening. Functional data were registered to their corresponding structural images and transformed to Montreal Neurological Institute (MNI) space using a reference brain (12 DOF linear transformation). Multivariate Exploratory Linear Optimized Decomposition into Independent Components (MELODIC) was used to remove artefacts (these comprised 2% of all MELODIC components).

### Analysis 1: effect of task

MELODIC filtered data were entered into a first-level analysis, to produce contrast of parameter estimate (COPE) images for blood oxygen level-dependent (BOLD) response to food and control images separately. Mean BOLD % signal change (unthresholded) was extracted with Featquery using masks of a priori regions of interest (ROI) selected from standard templates from WFUPickatlas (Maldjian et al. 2003). The ROIs chosen were based on previous neuroimaging work (see Supplemental Table 1 for list of ROIs and supporting references). The BOLD % signal change to food and control images was compared using paired sample *t* tests using IBM SPSS (version 23). Bonferroni correction was applied to control for the family-wise error (FWE).

### Analysis 2: effect of placebo versus mCPP on BOLD signals to high- and low-calorie foods

MELODIC filtered data were entered into a first-level analysis, to produce COPE images for high-calorie and low-calorie food images, minus the BOLD response to the corresponding control images. These COPEs were averaged across each of the scanning blocks for each participant. A subsequent analysis was run on these outputs for each participant to subtract baseline scans from post-dosing scans. The outputs were entered into the final mixed effects (FLAME 1 + 2) group analysis, producing contrasts between placebo and mCPP conditions for BOLD signal activity in response to the high-calorie food images and the low-calorie food images separately.

Group Z statistic images were corrected for multiple comparisons by FWE correction using AlphaSim, part of the AFNI toolkit (Cox 1996) (AFNI Version 16.1.16—May 25, 2016). With a voxel-wise threshold of *p* < 0.005 (*Z* > 2.6), only clusters with more than 24 contiguous voxels were significant with a FWE rate corrected *p* < 0.05.

#### Covariates

Analysis 2 above was repeated with mean centred VAS ratings (taken immediately prior to each scan) of nausea, light-headed and faint entered as covariates (separately) to account for any non-specific effects of mCPP on the BOLD response to food images.

### Data analysis

Main effects and interactions with condition were examined with analysis of variance (ANOVA). Bonferroni correction was used on all follow-up *t* tests unless otherwise stated.

#### Data loss

Data were lost for seven pasta sessions and two cookie sessions due to technical issues, such as participants leaning on the balance. In addition, one participant did not return for the second session.

## Results

### Participant characteristics

The sample comprised young women (mean age = 22.7 (SEM: 1.19)) with a lean BMI (mean BMI = 21.8 (SEM: 0.32)). TFEQ scores were as follows: cognitive restraint (mean = 5.77 (SEM: 0.49)), disinhibition (mean = 7.1 (SEM: 0.78)) and hunger (mean = 6.5 (SEM: 0.84)). Participant questionnaire scores on the BDI, BIS-11, BFS, PFS, PANAS and STAI are summarised in Supplemental Table 2.

### Salivary cortisol

Cortisol levels at baseline and immediately prior to the test meal were analysed with ANOVA: there were no main effects of condition (*F* (1 22) = 4.03; *p =* 0.06) or time (*F* (1 22) = 0.75; *p =* 0.4), but a significant interaction between condition and time (*F* (1 22) = 8.85; *p =* 0.007). There were no baseline differences between mCPP and placebo conditions (4.98 vs. 5.59 nmol/L; *t* (22) = − 1.14, *p* = 0.3); however, immediately prior to food, cortisol was significantly higher in the mCPP than the placebo condition (6.62 vs. 2.77 nmol/L; *t* (22) = − 2.62, *p* = 0.03).

### Appetite and mood ratings

Baseline VAS ratings did not differ according to condition (all *p*s > 0.05—data not shown). *t* tests on area under the curve scores (AUC—trapezoid method) showed increased scores in the mCPP condition relative to placebo for faint (2254.13 vs. 706.30; *t* (22) = − 3.05, *p* = 0.006), light-headed (2938.26 vs. 751.09; *t* (22) = − 5.09, *p* = 0.00004) and nausea (2158.04 vs. 606.09; *t* (22) = − 5.27, *p* = 0.00003) along with decreased hunger (6595.30 vs. 8172.39; *t* (22) = 2.11, *p* = 0.047) and desire to eat (6168.04 vs. 7830.65; *t* (22) = 2.10, *p* = 0.048). There were no other significant effects of condition nor any interactions (all *p* > 0.05) (Supplemental Table 3).

### UEM measures

#### Pasta

**Fig. 2:**
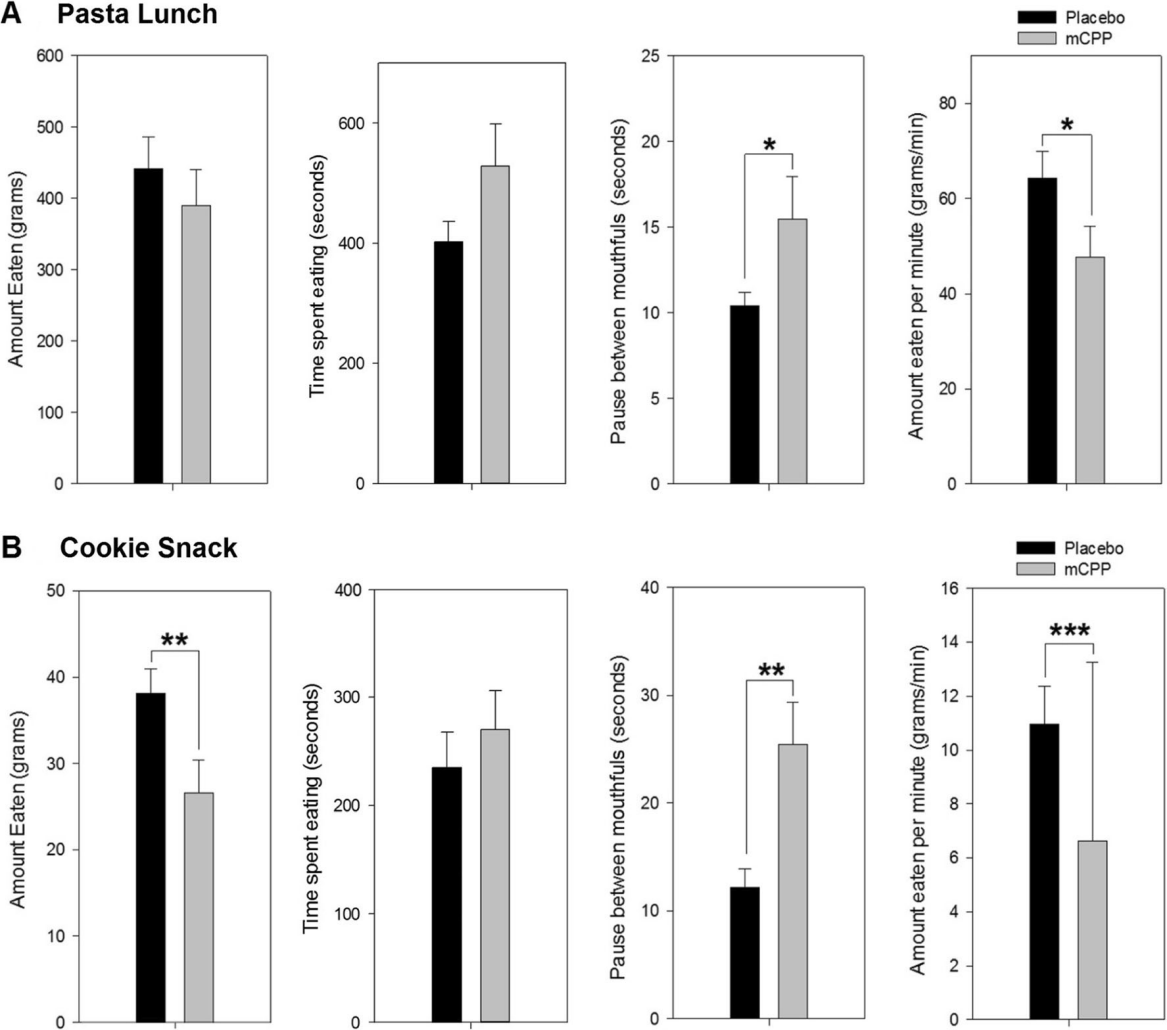
Universal eating monitor measures for the pasta lunch (**a**) and cookie snack (**b**) split by placebo and mCPP conditions. mCPP significantly reduced the consumption of cookies but not pasta. For both foods, mCPP significantly increased the pause between mouthfuls and reduced the eating rate (amount eaten per minute). Time spent eating was not significantly different for either food after dosing with mCPP. *p < 0.05; **p < 0.01; ***p < 0.001

Rate of pasta consumption was significantly reduced by mCPP (*t* (16) = 2.27, *p* = 0.04), and pauses between mouthfuls increased (*t* (16) = −2.32, *p* = 0.03, Fig. 2). There was no main effect of drug for total amount eaten (*t* (16) = 1.43, *p* = 0.2) or time spent eating (*t* (22) = 1.43, *p* = 0.2).

#### Cookies

In the mCPP condition, participants ate fewer cookies (*t* (20) = 3.09, *p* = 0.006), at a slower rate (*t* (18) = 4.12, *p* = 0.0004), and took longer pauses between mouthfuls compared with the placebo condition (*t* (18) = − 3.82, *p* = 0.001 (Fig. 2)). No significant differences were observed for total time spent eating (*t* (20) = − 0.98, *p* = 0.4).

#### Within-meal VAS pleasantness ratings

mCPP tended to reduce rated pleasantness of the pasta (*t* (16) = 1.99, *p* = 0.06; 68.79 mm for mCPP vs. 73.97 mm for placebo). The cookies were rated as less pleasant after mCPP relative to placebo (*t* (19) = 2.46, *p* = 0.02; 78.36 vs. 84.84 mm).

#### Correlations between hunger, nausea and intake

After mCPP, pasta intake was significantly positively correlated with hunger (*r* = 0.52, *n* = 19, *p* < 0.05); pasta intake was not correlated with either rated nausea, light-headedness or sensations of faint (all *p*s > 0.05). Cookie intake after mCPP did not correlate with hunger, nausea, light-headed nor faint (all *p*s > 0.05). In addition, including a composite measure of nausea, light-headed and faint ratings as a covariate in the analyses did not affect the pattern of results for consumption of pasta or cookies.

### fMRI

#### Main effect of task

The following regions showed a significantly greater BOLD response to food compared to control images: nucleus accumbens, midbrain, orbitofrontal cortex, ventromedial prefrontal cortex, insula, amygdala, cingulate cortex (anterior and posterior), dorsal striatum (caudate and putamen) and dorsolateral prefrontal cortex (inferior and middle frontal gyrus) (all *p*s < 0.05—Supplemental Table 1). Additional follow-up analysis comparing responses to high-calorie versus low-calorie pictures revealed no significant differences according to picture type (data not shown).

#### Placebo versus mCPP contrast

##### High-calorie foods

**Fig. 3:**
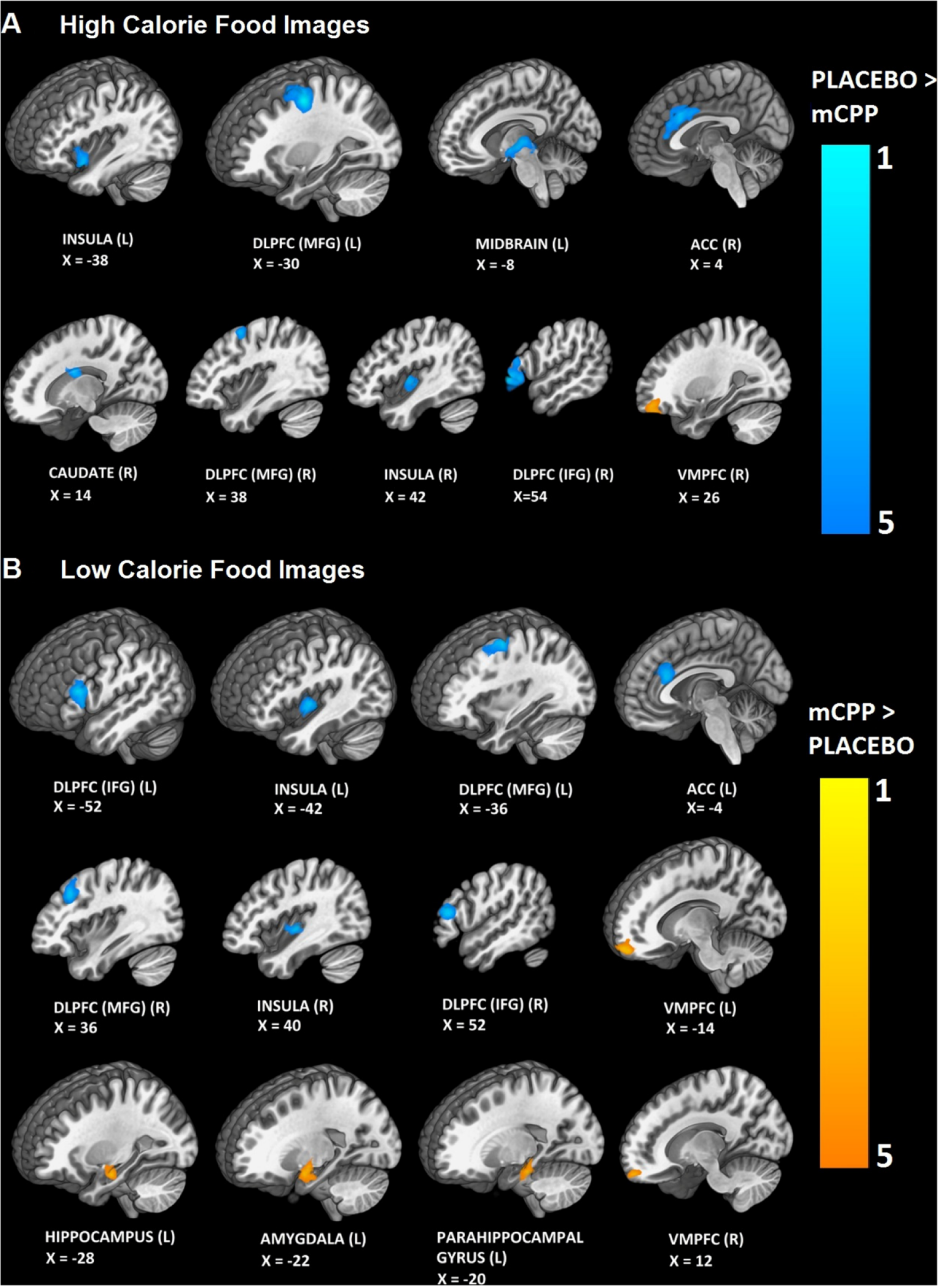
BOLD response to high- and low-calorie food images (**a** and **b**, respectively). Orange depicts brain areas where the BOLD response was greater after dosing with mCPP compared to placebo whereas blue depicts brain areas where participants show a greater BOLD response when dosed with placebo compared to mCPP. L left, R right, dlPFC dorsolateral prefrontal cortex, IFG inferior frontal gyrus, MFG middle frontal gyrus, ACC anterior cingulate cortex, IFG inferior frontal gyrus, vmPFC ventromedial prefrontal cortex

mCPP attenuated activity in the left dorsolateral prefrontal cortex (dlPFC), right dlPFC, right anterior cingulate cortex (ACC), left and right insula, right caudate and left midbrain, while increasing activity in the right ventromedial prefrontal cortex (vmPFC) (Fig. 3 and Table 1).

##### Low-calorie foods

mCPP attenuated activity in the left and right dlPFC, left and right insula, and left ACC, while increasing activity in the left and right vmPFC, left hippocampus, left parahippocampal gyrus and left amygdala (Fig. 3 and Table 1).

##### Covariates

Including either nausea, light-headed or faint ratings in the analyses did not affect either the direction or the significance of any of the local maxima reported here (*Z* scores for all local maxima remained equal to or greater than the threshold of *p* < 0.005).

#### Post hoc analysis: responders versus non-responders

Inspection of the intake data revealed that some participants ate less after mCPP but others showed no response, or an increase in intake. Participants were classified as responders if they showed a > 10% decrease in cookie consumption after mCPP versus placebo, and non-responders if they showed a < 10% decrease in consumption after mCPP versus placebo (12 responders and 8 non-responders). Analysis was conducted on cookie intake and restricted to responses to high-calorie food images because mCPP had a significant effect on cookie but not pasta intake.

#### Responder versus non-responder characteristics

Responders and non-responders did not differ in terms of basic characteristics (age, BMI and TFEQ subscales) or baseline questionnaire and VAS scores averaged across both test sessions (all *p*s > 0.05—see Supplemental Table 4). While pre-cookie hunger ratings were not significantly different between responders and non-responders (21.6 vs. 21.1 mm; *t* (16) = − 0.05, *p* > 0.05), the non-responder group rated cookies as significantly more pleasant than the responder group (85.3 vs. 70.9 mm; (*t* (16) = 2.27, *p* < 0.05).

#### Baseline BOLD responses

**Fig. 4:**
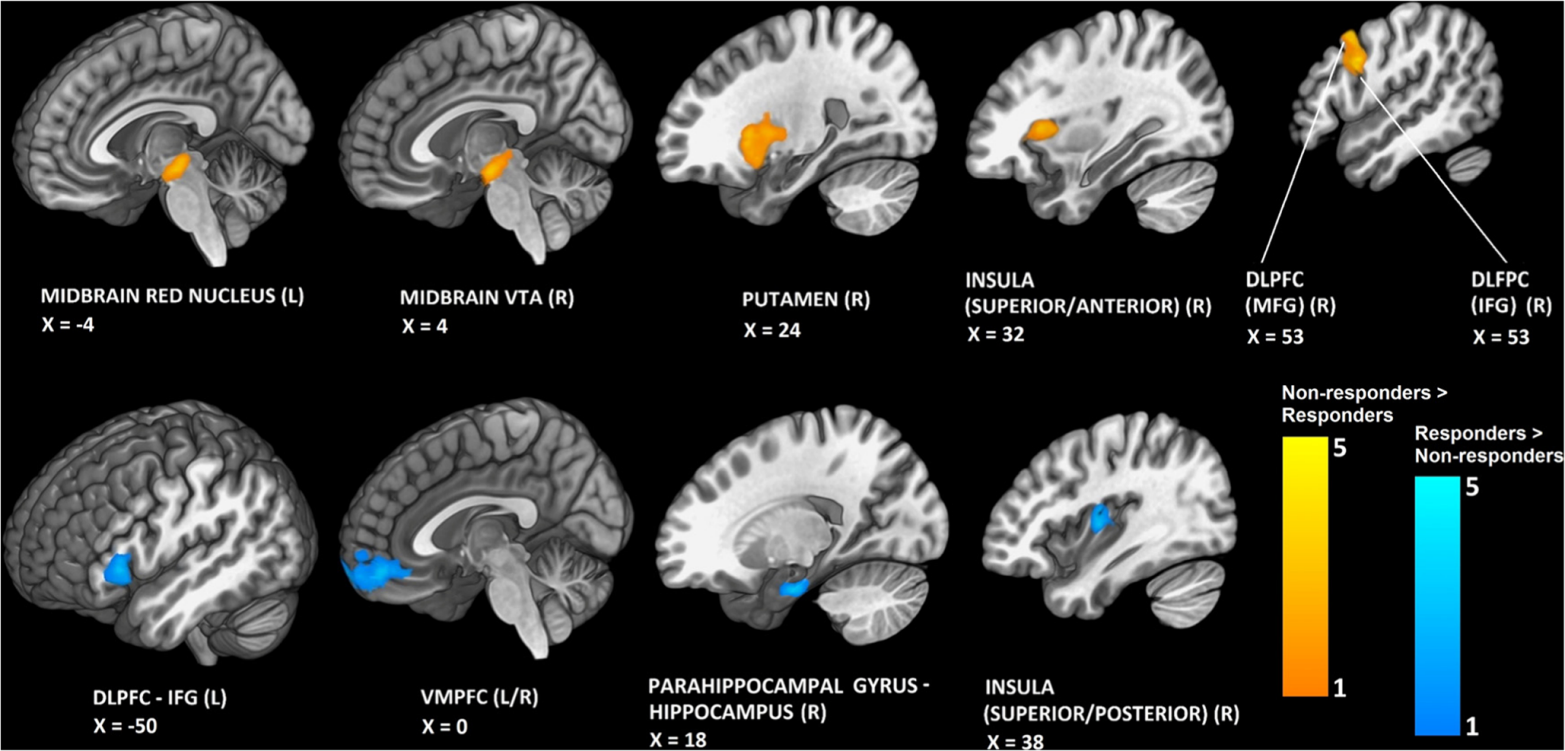
Baseline BOLD response to high-calorie food images. Orange depicts brain areas where non-responders show a greater BOLD response than responders; blue depicts brain areas where responders show a greater BOLD response than non-responders. L left, R right, VTA ventral tegmental area, dlPFC dorsolateral prefrontal cortex, MFG middle frontal gyrus, IFG inferior frontal gyrus, vmPFC ventromedial prefrontal cortex

Baseline scan data were pre-processed as described above in analysis 1. The data from pre-mCPP and pre-placebo baseline scans were averaged. The outputs were entered into a mixed effects model, to produce contrasts between non-responders and responders for BOLD signal activity in response to high-calorie foods. Non-responders showed a greater BOLD response than responders in the right dorsolateral prefrontal cortex, right brain stem, left brain stem, right insula and right putamen. Responders showed a greater response than non-responders in the vmPFC, right insula and left dorsolateral prefrontal cortex (Fig. 4; local maxima in Table 1).

**Table 1:** Local maxima of key appetitive and reward areas showing (A) main effect of condition (placebo vs. mCPP), split by activity to high- and low-calorie food images and (B) differences in BOLD signal between non-responders and responders at baseline to the sight of high-calorie food images

Brain region (hemisphere)	Montreal Neurological Institute (MNI) coordinates			Brodmann area	*Z* score
	*X*	*Y*	*Z*		
A. Main effect of condition					
Reduced activation after mCPP (compared to placebo)					
High-calorie food images					
dlPFC (middle frontal gyrus)/precentral gyrus (L)	− 30	− 6	54	6	5.2
Anterior cingulate rortex (R)	4	22	30	24	3.3
Insula (L)	− 38	10	− 14	48	3.3
Caudate (R)	14	− 2	24	–	3.1
dlPFC (middle frontal gyrus) (R)	38	6	58	6	3.0
dlPFC (inferior frontal gyrus) (R)	54	28	0	45	2.8
Insula (R)	42	− 10	0	48	2.8
Midbrain (L)	− 8	− 14	− 4	–	2.7
Low-calorie food images					
dlPFC (inferior frontal gyrus) (L)	− 52	26	20	45	3.9
dlPFC (inferior frontal gyrus) (R)	52	32	16	48	3.9
dlPFC (middle frontal gyrus) (L)	− 36	− 2	62	6	3.6
dlPFC (middle frontal gyrus) (R)	36	26	42	9	3.5
Insula (L)	− 42	− 8	2	48	3.4
Insula (R)	40	− 14	0	48	3.3
Anterior cingulate cortex (L)	− 4	26	28	24	3.1
Increased activation after mCPP (compared to placebo)					
High calorie					
Ventromedial prefrontal cortex (R)	26	52	− 12	11	3.1
Low calorie					
vmPFC (L)	− 14	62	− 16	11	3.3
Parahippocampal gyrus (L)	− 20	− 26	− 22	30	3.2
Hippocampus (L)	− 28	− 14	− 20	20	3.2
Amygdala (L)	− 22	− 6	− 18	34	3.1
vmPFC (R)	12	60	− 18	11	2.8
B. Responders versus non-responders					
Non-responders > responders					
Dorsolateral prefrontal cortex (inferior frontal gyrus) (R)	52	12	30	44	4.4
Dorsolateral prefrontal cortex (middle frontal gyrus) (R)	54	14	44	44	4.0
Brainstem (midbrain–ventral tegmental area) (R)	4	− 16	− 14	–	3.6
Brainstem (midbrain–red nucleus) (L)	− 4	− 20	− 12	–	3.5
Insula (superior/anterior) (R)	32	22	8	48	3.4
Putamen (R)	24	14	0	48	3.1
Responders > non-responders					
Ventromedial prefrontal cortex	0	54	− 12	11	3.7
Insula (superior/posterior) (R)	38	− 8	6	48	3.6
Dorsolateral prefrontal cortex (inferior frontal gyrus) (L)	− 50	26	2	45	3.6
Parahippocampal gyrus/hippocampus (R)	18	− 10	− 26	28	3.5

FWE cluster corrected (voxel *p* < 0.005; cluster > 24 contiguous voxels—*p* < 0.05)

*L* left side, *R* right side

## Discussion

The 5-HT_2C_ receptor agonist mCPP increased salivary cortisol confirming activation of 5-HT_2C_ receptors. mCPP decreased intake of cookies eaten in the absence of hunger but had no effect on the amount of a pasta lunch consumed, although the pasta eating rate was reduced. mCPP also decreased BOLD responses to the sight of food pictures in reward-associated circuitry. These data suggest a role for 5-HT_2C_ receptor activation in mediating reward-related responses to food in humans.

Consistent with the present findings, Thomas et al. (2014) reported that mCPP did not reduce pasta intake but reduced rated appetite. By contrast, mCPP reduced intake of cookies and blunted the rated pleasantness of cookies. The effect of mCPP on microstructural measures of eating was also greater for cookie than for pasta consumption. Thirty milligrams of mCPP reduced the rate of pasta consumption by 26% and cookie consumption by 39% and increased the duration of pauses between mouthfuls of pasta and cookies by 49 and 109%, respectively. Taken together, these data suggest that the effect of mCPP on appetite was greater for the cookies than for the pasta.

mCPP increased ratings of nausea-like symptoms but these ratings were not correlated with food intake, suggesting they were unlikely to account for the effects on eating behaviour, as shown previously (Walsh et al. 1994; Thomas et al. 2014). In addition, the differential effects of mCPP on pasta and cookie intake suggest that these effects are not secondary to nausea which would be expected to suppress intake of both foods to a similar extent.

It is not possible to conclude whether the effects of mCPP were related to the greater palatability of the cookies, their greater energy density or both. Indeed, there are several differences between the pasta and cookies, including their sensory characteristics that might explain the pattern of results. In addition, the cookies were served after a satiating meal, and it may be that the effects of mCPP are enhanced under conditions of satiety. 5-HT_2C_ receptors may mediate reductions in food reward that occur as food is consumed, known as alliesthesia (Cabanac 1971). It will be important to test this hypothesis because reductions in reward-related responding that are specific to the satiated state are likely to be effective in helping individuals to curb their appetite but are unlikely to reduce hedonic responding in general as observed for the withdrawn anti-obesity drug rimonabant (Butler and Korbonits 2009). Such investigations are also likely to shed light on the role of background neural activity and experimental context in the effects of 5-HT_2C_ receptor stimulation on reward-related behaviour (Vollm et al. 2010).

mCPP attenuated BOLD activity to the sight of both high and low food images in a number of brain regions involved in reward including the insula, anterior cingulate cortex, dorsolateral prefrontal cortex and caudate (Wang et al. 2008; Holroyd and Yeung 2012; Morris et al. 2014). This pattern of results was not affected by adding nausea-like ratings as a covariate in the analysis suggesting that the results are not explained by negative side effects of the drug. 5-HT_2C_ receptors are located in the prefrontal cortex, cingulate cortex and the caudate (Pazos et al. 1987; Pompeiano et al. 1994; Marazziti et al. 1999), suggesting that mCPP may act in these areas to affect responding to food stimuli. Areas showing increased activation after mCPP versus placebo were limited to the vmPFC, hippocampus, parahippocampal gyrus and amygdala. It has been proposed that activity in the vmPFC and dlPFC is associated with context-dependent value-based decision-making and selective attention to motivationally relevant stimuli and the pattern of BOLD activity observed could suggest that the influence of contextual factors, such as metabolic state, on food valuation is altered by mCPP (Rudorf and Hare 2014; Walton et al. 2015). Since we found a stronger anorectic response after mCPP when eating in the absence of hunger, it would be of interest to examine whether the effect of mCPP on BOLD responses is dependent upon levels of satiety.

There was no effect of mCPP on hypothalamic responses but we draw no strong conclusion about this null effect because the hypothalamus is difficult to image and is susceptible to artefacts due to its proximity to the sinuses (Ojemann et al. 1997), which might also explain the lack of a main effect of task for hypothalamic responding. A recent study of the effects of lorcaserin on BOLD responses to food pictures in participants with obesity similarly failed to find an effect on hypothalamic activation (Farr et al. 2016). However, participants who received lorcaserin twice a day showed less activation in insula, parietal cortex, visual cortices, hippocampus and amygdala in the fasting state at 1 than 4 weeks. Although the study of Farr et al. (2016) is not directly comparable to the present study, due to several methodological differences between the studies, the data suggest that there is some overlap between the effects of mCPP and lorcaserin on responding to food pictures in the short term, which could point towards common 5-HT_2C_ receptor-mediated effects.

An exploratory post hoc analysis examined apparent individual variability in response to mCPP. At baseline, participants who did not respond to mCPP by decreasing their intake of cookies showed greater BOLD activity than responders in areas including dorsolateral prefrontal cortex, insula and putamen and midbrain. After dosing with mCPP, non-responders rated cookies as more pleasant than responders, in the absence of differences in rated hunger suggesting that heightened reward response might be responsible for blunting the hypophagic effect of mCPP. Further investigation of the characteristics of responders versus non-responders is required, but our results suggest that imaging data may shed light on which individuals are likely to show reduced food intake after treatment with 5-HT_2C_ receptor agonists.

We provide the first evidence that mCPP reduces consumption of a palatable energy dense snack in humans. mCPP also caused a marked reduction in neural activity across reward-related brain regions to the sight of food. An implication of these findings is that 5-HT_2C_ receptor agonists such as lorcaserin may be effective in helping individuals to reduce their intake of palatable food in the absence of hunger. In addition, we found that some participants did not reduce their cookie intake after treatment with mCPP and this was associated with enhanced rated cookie pleasantness and enhanced baseline BOLD responses to food in key reward areas. Further investigation of stratification of responding to mCPP (and potentially other 5-HT_2C_ receptor agonists such as lorcaserin) is required to identify patients who are more likely to respond to weight management drugs that act at the 5-HT_2C_ receptor and hence more effectively target therapy.

## Electronic supplementary material

ESM 1 (DOCX 1717 kb)
