# Supplementary material for: The 5-HT2C receptor agonist meta-chlorophenylpiperazine (mCPP) reduces palatable food consumption and BOLD fMRI responses to food images in healthy female volunteers
Source: Psychopharmacology (Berl). 2017 Oct 28;235(1):257–67. doi: 10.1007/s00213-017-4764-9 (PMC5748416; doi:10.1007/s00213-017-4764-9)
Supplement: Supplementary file 1 — (DOCX 1717 kb) [file 213_2017_4764_MOESM1_ESM.docx]

**Title: The 5-HT_2C_ receptor agonist meta-chlorophenylpiperazine (mCPP) reduces palatable food consumption and BOLD fMRI responses to food images in healthy female volunteers.**

**Running title: 5-HT_2C_ receptors and food reward**

Jason M. Thomas PhD^1,2^, Colin T. Dourish DSc^3^, Jeremy Tomlinson PhD^4^, Zaki Hassan-Smith PhD^5^, Peter C. Hansen DPhil^1^ & Suzanne Higgs PhD^1^.

^1^School of Psychology, University of Birmingham, Edgbaston, Birmingham, B15 2TT,

^2^Department of Psychology, Aston University, Birmingham, UK, B4 7ET.

^3^P1vital, Manor House, Howbery Park, Wallingford, Oxfordshire OX10 8BA,

^4^Oxford Centre for Diabetes, Endocrinology and Metabolism, Oxford NIHR Biomedical Research Centre, University of Oxford, Churchill Hospital, Headington OX7 3LJ,

^5^Centre for Endocrinology, School of Clinical and Experimental Medicine, University of Birmingham, Birmingham B15 2TH.

All correspondence and reprint requests to be directed to Suzanne Higgs.

Mailing Address: School of Psychology, University of Birmingham, Birmingham, B152TT.

Email: s.higgs.1@bham.ac.uk

Telephone: +44 (0)121 4144907

**Abstract**

Brain 5-HT_2C_ receptors form part of a neural network that controls eating behaviour. 5-HT_2C_ receptor agonists decrease food intake by activating proopiomelanocortin (POMC) neurons in the arcuate nucleus of the hypothalamus, but recent research in rodents has suggested that 5-HT_2C_ receptor agonists may also act via dopaminergic circuitry to reduce the rewarding value of food and other reinforcers. No mechanistic studies on the effects of 5-HT_2C_ agonists on food intake in humans have been conducted to date. The present study examined the effects of the 5-HT_2C_ receptor agonist meta-chlorophenylpiperazine (mCPP) on food consumption, eating microstructure and Blood Oxygen Level Dependent (BOLD) functional Magnetic Resonance Imaging (fMRI) responses to food pictures in healthy female volunteers. mCPP decreased rated appetite and intake of a palatable snack eaten in the absence of hunger but had no significant effect on the consumption of a pasta lunch (although pasta eating rate was reduced). mCPP also decreased BOLD fMRI responses to the sight of food pictures in areas of reward-associated circuitry. A post-hoc analysis identified individual variability in the response to mCPP (exploratory responder-non-responder analysis). Some participants did not reduce their cookie intake after treatment with mCPP and this lack of response was associated with enhanced ratings of cookie pleasantness and enhanced baseline BOLD responses to food images in key reward and appetite circuitry. These results suggest that 5-HT_2C_ receptor activation in humans inhibits food reward-related responding and that further investigation of stratification of responding to mCPP and other 5-HT_2C_ receptor agonists is warranted.

**Introduction**

Recent work on the neurophysiological basis of eating behaviour suggests that there are close interactions between the homeostatic networks that respond to changes in metabolic state and those involved in assigning reward value to motivational stimuli and translating motivation into action (Berthoud, 2011). For example, food deprivation increases the incentive value of food, which is reflected in enhanced responses to appetitive stimuli in reward-related brain areas whereas satiation decreases responses in reward-related circuitry (Goldstone et al., 2009, Thomas et al., 2015; see van der Lann et al., 2010 for a meta-analysis). These effects are likely to be mediated by the action of metabolic signals such as leptin, insulin, peptide YY (PYY) and ghrelin on the activity of the mesocorticolimbic dopamine system (Batterham, et al., 2007; Guthoff, et al., 2010; Farooqi, et al., 2007; Malik et al., 2008).

The role of serotonin in the control of appetite has been largely interpreted within a framework of homeostatic eating and the influence of hypothalamic cellular mechanisms in the effects of serotonergic drugs on food intake is well documented. The melanocortin system of the arcuate nucleus of the hypothalamus (ARC) has been identified as a key network in the anorectic effects of serotonin agonists, including the 5-HT_2C_ receptor agonist lorcaserin, which has recently been approved by the FDA to treat obesity (Heisler et al., 2002, 2006; Sohn et al., 2011). However, alterations in serotonin transmission also affect reward-related circuits in the brain to influence food intake, either directly (Aronson et al., 1995) or indirectly via modulation of dopamine activity. Indeed, 5-HT_2C_ receptors expressed in the ventral tegmental area (VTA) (Bubar, & Cunningham, 2007) modulate activity of dopaminergic (DA) projections to the nucleus accumbens (NAcc) to alter motivation for food and drug reinforcers in rats (Fletcher et al., 2004; Higgins et al., 2013). These preclinical data suggest a specific role for 5-HT_2C_ receptor activation in linking hypothalamic energy-sensing mechanisms to motivational aspects of eating behaviour. However, to date, no mechanistic studies on the effects of 5-HT_2C_ receptor agonists on food intake in humans have been reported.

Our aim here is to test the hypothesis that 5-HT_2C_ receptor activation reduces food-reward responses in humans. We investigate for the first time the effect of the preferential 5-HT_2C_ receptor agonist, meta-chlorophenylpiperazine (mCPP) on hedonic eating and neural responses to food images using functional Magnetic Resonance Imaging (fMRI). mCPP is known to reduce appetite in humans (Cowen et al. 1995; Walsh et al., 1994; Thomas et al., 2014) but to probe reward-related effects of mCPP we examine the effect of mCPP on the consumption of a staple meal consumed in a hungry state and a palatable high energy dense snack food eaten in the absence of hunger. We also examine the microstructure of eating using a Universal Eating Monitor (UEM) to identify specific behavioural changes that may underlie decreases in food intake (Thomas et al., 2014). We predicted that mCPP would reduce rated appetite (Thomas et al., 2014), but that the drug effect on intake would be greater for the palatable food consumed in the absence of hunger than for the staple food consumed when hungry. We also predicted that this effect of mCPP on palatable food consumption might be mediated by a reduction in rated pleasantness of the food and reflected in a reduction of markers of neural activation in reward-related brain circuitry.

**Materials & Methods**

*Participants*

24 women volunteers were recruited from the University of Birmingham. Posters advertised the study as an “Appetite & fMRI study”, and participants were compensated with cash or course credits upon completion. The sample size was based on the results of a previous study (Thomas et al. 2014). Ethical approval was provided by South Birmingham Research Ethics Committee (National Research Ethics Service number 11/WM/0411) and informed consent was provided by all participants. Participants were screened to exclude the following: under 18 or over 65 years old; body mass index (BMI) under 18.5 or over 24.9 kg/m^2^; English not first language; taking any psychotropic medication or recreational drugs; past or current Axis 1 disorder (determined by the Structured Clinical Interview for DSM-IV Axis I Disorders; SCID-I/P; Spitzer et al., 2004); pregnant or breastfeeding; smoker; dyslexic; food allergies; diabetic; cognitive dietary restraint score higher than 10 as measured by the Three-Factor Eating Questionnaire (TFEQ; Stunkard and Messick 1985). Participants were also excluded if they had previously taken part in a mCPP study, were left-handed, or had any contraindications to fMRI scanning. Women were asked to participate in test days that fell outside their premenstrual week.

*Design*

In a double-blind, placebo-controlled, crossover design, participants were randomized immediately after the screening days to receive oral mCPP (30mg) (Thomas et al., 2014) in a single morning dose, or placebo, in a counterbalanced order. mCPP and the matched placebo were supplied by the Guy’s and St Thomas’ NHS Foundation Trust Pharmacy Manufacturing Unit. The washout period between test sessions was 7 days. To maintain blinding, mCPP and placebo were prepared in identical capsules and unblinding occurred on study completion. Peak plasma levels of mCPP are observed 120-180 min after oral administration, which was timed to coincide with the second fMRI scan.

*Universal Eating Monitor (UEM)*

Food was served on a UEM consisting of a balance (Sartorius Model CP4201, Sartorius Ltd., Epsom, UK; 0.1g accuracy) placed underneath the surface of a table and connected to a laptop computer. A placemat hid the balance from view (Thomas et al., 2014).

*Pasta:* Dishes filled with 220g of pasta were provided. Each time the participant ate 50g of pasta, the Sussex Ingestion Pattern Monitor (SIPM) software (version 2.0.13) interrupted the participant to complete computerised VAS ratings (hunger, fullness and pleasantness of the pasta). After consuming 150g, participants were interrupted and provided with a fresh dish of 220g of pasta. Participants were asked to eat in this manner until they felt ‘comfortably full’. The lunch consisted of pasta shells in a tomato and herb sauce served at 55-60^o^C (207kcal per 220g serving).

*Cookies:* Bowls containing 80g of cookie pieces were provided. Each time the participant ate 10g of cookie pieces, the SIPM software interrupted the participant to complete VAS ratings as described above. After consuming 60g, participants were interrupted and provided with a fresh bowl containing 80g of cookie pieces. Participants were asked to eat until they felt ‘comfortably full’. The cookies were Maryland Chocolate Chip Cookies, with each cookie being broken into 6-7 pieces (390kcal per 80g serving).

*Salivary cortisol assessment*

Salivary cortisol was collected to confirm a pharmacological response to mCPP administration (Meltzer and Maes, 1995) and was measured by liquid chromatography–mass spectrometry (LC–MS/MS) as described previously (Thomas et al., 2014).

*Procedure*

The experimental procedure is summarised in Figure 1.

**INSERT FIGURE 1**

*Screening Days:* Participants who met the study criteria were invited to a screening day at which they completed: a medical screening sheet; the Eysenck Personality Questionnaire (EPQ; Eysenck and Eysenck 1975); and a questionnaire to determine whether they usually consume lunch. Height and weight were also taken to calculate BMI. Participants returned after a week for two practice sessions, both a week apart, with the UEM.

*Test Days:* Participants arrived having consumed their normal breakfast. If they passed a medical examination (first day only), they were breathalysed and completed a pregnancy test, before completing the first batch of questionnaires to assess what and when they had eaten that morning, and several measures to assess mood: Beck Depression Inventory (BDI; Beck et al.,1961); Befindlichskeit Scale of mood and energy (BFS; von Zerssen et al., 1974); Positive and Negative Affect Schedule (PANAS; Watson et al., 1988); and State-Trait Anxiety Inventory (STAI; Spielberger 1983). They also completed the Power of food Scale (PFS, Lowe et al., 2009), and the Barratt Impulsivity Scale (BIS – Patton et al., 1995) and baseline visual analogue scale (VAS) to assess: ‘alertness’; ‘disgust’; ‘drowsiness’; ‘light-headed’; ‘anxiety’, ‘happiness’; ‘nausea’; ‘sadness’; ‘withdrawn’; ‘faint’; ‘hungry’; ‘full’; ‘desire to eat’; and ‘thirst’.

A baseline fMRI scan was then conducted, after which participants completed the VAS, provided a saliva sample, and took either mCPP or placebo. At 30 minutes post-dosing they completed another set of VAS. 30 minutes later, participants provided a saliva sample and completed VAS. They were scanned again and then completed a set of VAS and provided another saliva sample.

Immediately before lunch, participants completed VAS and were given ad-libitum access to a pasta lunch via the UEM. After lunch, participants completed VAS followed by a 20 minute break, after which a further set of VAS was completed before participants were given ad-libitum access to a cookie snack. Immediately after the snack, participants filled out VAS. Approximately 30-40 minutes later participants completed a second batch of questionnaires: VAS; BDI; BFS; PANAS; STAI; PFS; and BIS and provided a final saliva sample, rated the scanner task food images and had a single blood sample taken. At the end of their second session participants were fully debriefed, thanked for their time, and reimbursed for participation.

*Imaging task*

The scanner task was based on that used by Allen et al., 2016. There were three separate blocks in which participants viewed 40 food pictures (20 high calorie food images and 20 low calorie food images), 40 non-food control pictures and 5 smiley face images in three separate blocks. The high calorie images (mean number of calories per 100g of food shown = 365) and low calorie images (mean number of calories per 100g of food shown = 79) were comparable with previously published tasks (Goldstone et al., 2009). The pictures were displayed for 2500ms, fixation points were displayed for 3500ms, and smiley face images were displayed for 1500ms. Participants were asked to pay attention to all images, but to imagine eating the foods they saw during the task, and to press a button on a button box when they saw a smiley face to ensure they were maintaining attention.

*fMRI data acquisition and analysis*

An event-related design was used, in which the stimuli were presented in pseudo random sequence. The scanner was a 3.0 T Achieva (Philips) whole body scanner with an 8 channel head coil. T2*weighted echo planar imaging (EPI) slices were acquired every 2.5 seconds (TR = 2.5). 33 axial slices with an (in-plane resolution of 2.5 x 2.5 x 3 mm2 and slice thickness 3mm ( - no gap) were acquired, with a matrix size of 96 x 96 and field of view of 240 x 240 mm. Acquisition angulation was consistently AC-PC. 200 volumes were acquired for each block of the task with two dummy scans which were discarded prior to any analysis. A whole brain T2*-weighted EPI volume (resting state) was also acquired (AC-PC angulation), along with an anatomic T1-weighted volume acquired in the: sagittal plane slice thickness of 1 mm and in-plane with a reconstructed resolution of 1.0 x 1.0 x 1.0 mm. The FMRIB software library (FSL; FMRIB, Oxford, www.fmrib.ox.ac.uk/fsl) was used for pre-processing and data analyses. Pre-processing involved: high pass filter cut off of 60s; motion correction using FMRIB's Linear Image Registration Tool (MCFLIRT); motion parameters as regressors of no interest; interleaved slice timing correction; spatial smoothing with a 6mm full-width-half-maximum kernel; high pass temporal filtering and FILM pre-whitening. Functional data were registered to their corresponding structural images and transformed to Montreal Neurological Institute (MNI) space using a reference brain (12 DOF linear transformation). Multivariate Exploratory Linear Optimized Decomposition into Independent Components (MELODIC) was used to remove artefacts (these comprised 2% of all MELODIC components).

*Analysis 1: Effect of task*

MELODIC filtered data were entered into a first level analysis, to produce contrast of parameter estimate (COPE) images for Blood Oxygen Level Dependent (BOLD) response to food and control images separately. Mean BOLD % signal change (unthresholded) was extracted with Featquery using masks of a priori regions of interest (ROI) selected from standard templates from WFUPickatlas (Maldjian et al., 2003). The ROIs chosen were based on previous neuroimaging work (see Supplemental Table 1 for list of ROIs and supporting references). The BOLD % signal change to food and control images was compared using paired samples t-tests using IBM SPSS (Version 23). Bonferroni correction was applied to control for the familywise error (FWE).

*Analysis 2: Effect of placebo versus mCPP on BOLD signals to high and low calorie foods*

MELODIC filtered data were entered into a first level analysis, to produce COPE images for high calorie and low calorie food images, minus the BOLD response to the corresponding control images. These COPES were averaged across each of the scanning blocks for each participant. A subsequent analysis was run on these outputs for each participant to subtract baseline scans from post-dosing scans. The outputs were entered into the final mixed effects (FLAME 1+2) group analysis, producing contrasts between placebo and mCPP conditions for BOLD signal activity in response to the high calorie food images and the low calorie food images separately.

Group Z statistic images were corrected for multiple comparisons by FWE correction using AlphaSim, part of the AFNI toolkit (Cox, 1996) (AFNI Version 16.1.16 – May 25 2016). With a voxel-wise threshold of *p* < 0.005 (Z > 2.6), only clusters with more than 24 contiguous voxels were significant with a FWE rate corrected *p* < 0.05.

*Covariates:* Analysis 2 above was repeated with mean centered VAS ratings (taken immediately prior to each scan) of nausea, light-headed and faint entered as covariates (separately) to account for any non-specific effects of mCPP on the BOLD response to food images.

*Data Analysis*

Main effects and interactions with condition were examined with analysis of variance (ANOVA). Bonferroni correction was used on all follow-up t-tests unless otherwise stated.

*Data loss:* Data were lost for 7 pasta sessions and 2 cookie sessions due to technical issues, such as participants leaning on the balance. In addition, one participant did not return for the second session.

**Results**

*Participant characteristics*

The sample comprised young women (mean age = 22.7 (SEM: 1.19)) with a lean BMI (mean BMI = 21.8 (SEM: 0.32)). TFEQ scores were as follows: cognitive restraint (mean = 5.77 (SEM: 0.49)), disinhibition (mean = 7.1 (SEM: 0.78)) and hunger (mean = 6.5 (SEM: 0.84)). Participant questionnaire scores on the BDI, BIS-11, BFS, PFS, PANAS and STAI are summarised in Supplemental Table 2.

*Salivary Cortisol*

Cortisol levels at baseline and immediately prior to the test meal were analysed with ANOVA: there were no main effects of condition (*F* (1 22) = 4.03; *p =* 0.06), or time (*F* (1 22) = 0.75; *p =* 0.4), but a significant interaction between condition and time (*F* (1 22) = 8.85; *p =* 0.007). There were no baseline differences between mCPP and placebo conditions (4.98 versus 5.59nmol/L; *t* (22) = -1.14, *p* = 0.3), however, immediately prior to food, cortisol was significantly higher in the mCPP than the placebo condition (6.62 versus 2.77nmol/L; *t* (22) = -2.62, *p* = 0.03).

*Appetite and mood ratings*

Baseline VAS ratings did not differ according to condition (all *p*s > 0.05 – data not shown). T-tests on Area Under The Curve scores (AUC - trapezoid method) showed increased scores in the mCPP condition relative to placebo for faint (2254.13 vs. 706.30; *t* (22) = -3.05, *p* = 0.006), light-headed (2938.26 vs. 751.09; *t* (22) = -5.09, *p* = 0.00004) and nausea (2158.04 vs. 606.09; *t* (22) = -5.27, *p* = 0.00003) along with decreased hunger (6595.30 vs. 8172.39; *t* (22) = 2.11, *p* = 0.047) and desire to eat (6168.04 vs. 7830.65; *t* (22) = 2.10, *p* = 0.048). There were no other significant effects of condition nor any interactions (all *p* > 0.05) (Supplemental Table 3).

*UEM measures*

*Pasta:* Rate of pasta consumption was significantly reduced by mCPP (*t* (16) = 2.27, *p* = 0.04) and pauses between mouthfuls increased (*t* (16) = -2.32, *p* = 0.03, Figure 2). There was no main effect of drug for total amount eaten (*t* (16) = 1.43, *p* = 0.2), or time spent eating (*t* (22) = 1.43, *p* = 0.2).

*Cookies:* In the mCPP condition participants ate fewer cookies (*t* (20) = 3.09, *p* = 0.006), at a slower rate (*t* (18) = 4.12, *p* = 0.0004), and took longer pauses between mouthfuls compared with the placebo condition (*t* (18) = -3.82, *p* = 0.001, Figure 2). No significant differences were observed for total time spent eating (*t* (20) = -0.98, *p* = 0.4).

**INSERT FIGURE 2**

*Within-Meal VAS Pleasantness Ratings:* mCPP tended to reduce rated pleasantness of the pasta (*t* (16) = 1.99, *p* = 0.06; 68.79mm for mCPP versus 73.97mm for placebo). The cookies were rated as less pleasant after mCPP relative to placebo (*t* (19) = 2.46, *p* = 0.02; 78.36 versus 84.84mm).

*Correlations between hunger, nausea and intake:* After mCPP, pasta intake was significantly positively correlated with hunger (r = 0.52, n = 19, *p* < 0.05); pasta intake was not correlated with either rated nausea, light-headedness or sensations of faint (all *p*s > 0.05). Cookie intake after mCPP did not correlate with hunger, nausea, light-headed nor faint (all *p*s > 0.05). In addition, including a composite measure of nausea, light-headed and faint ratings as a covariate in the analyses did not affect the pattern of results for consumption of pasta or cookies.

*fMRI*

*Main effect of task*

The following regions showed a significantly greater BOLD response to food compared to control images: nucleus accumbens, midbrain, orbitofrontal cortex, ventromedial prefrontal cortex, insula, amygdala, cingulate cortex (anterior and posterior), dorsal striatum (caudate and putamen) and dorsolateral prefrontal cortex (inferior and middle frontal gyrus) (all *p*s < 0.05 –Supplemental Table 1). Additional follow up analysis comparing responses to high calorie versus low calorie pictures revealed no significant differences according to picture type (data not shown).

*Placebo versus mCPP contrast*
*High Calorie Foods*: mCPP attenuated activity in the left dorsolateral prefrontal cortex, right dlPFC, right anterior cingulate cortex (ACC), left and right insula, right caudate and left midbrain, while increasing activity in the right ventromedial prefrontal cortex (vmPFC) (Figure 3 and Table 4).

*Low Calorie Foods*: mCPP attenuated activity in the left and right dlPFC, left and right insula, and left ACC, while increasing activity in the left and right vmPFC, left hippocampus, left parahippocampal gyrus and left amygdala (Figure 3 and Table 4).

*Covariates:* Including either nausea, light-headed or faint ratings in the analyses did not affect either the direction or the significance of any of the local maxima reported here (Z scores for all local maxima remained equal to or greater than the threshold of *p* < 0.005).

**INSERT TABLE 1**

**INSERT FIGURE 3**

*Post-hoc analysis: Responders versus Non-responders*

Inspection of the intake data revealed that some participants ate less after mCPP but others showed no response, or an increase in intake. Participants were classified as responders if they showed a > 10% decrease in cookie consumption after mCPP versus placebo, and non-responders if they showed a < 10% decrease in consumption after mCPP versus placebo (12 responders and 8 non-responders). Analyses was conducted on cookie intake and restricted to responses to high calorie food images because mCPP had a significant effect on cookie but not pasta intake.

*Responder versus non-responder characteristics*

Responders and non-responders did not differ in terms of basic characteristics (age, BMI and TFEQ subscales) or baseline questionnaire and VAS scores averaged across both test sessions (all *p*s > 0.05 – see Supplemental Table 4). While pre-cookie hunger ratings were not significantly different between responders and non-responders (21.6mm versus 21.1mm; *t* (16) = -0.05, *p* > 0.05), the non-responder group rated cookies as significantly more pleasant than the responder group (85.3mm versus 70.9mm; (*t* (16) = 2.27, *p* < 0.05).

*Baseline BOLD responses*

Baseline scan data were pre-processed as described above in analysis 1. The data from pre-mCPP and pre-placebo baseline scans were averaged. The outputs were entered into a mixed effects model, to produce contrasts between non-responders and responders for BOLD signal activity in response to high calorie foods. Non-responders showed a greater BOLD response than responders in the right dorsolateral prefrontal cortex, right brainstem, left brain stem, right insula and right putamen. Responders showed a greater response than non-responders in the vmPFC, right insula and left dorsolateral prefrontal cortex (Figure 4; local maxima in Table 1).

**INSERT FIGURE 4**

**Discussion**

The 5-HT_2C_ receptor agonist mCPP increased salivary cortisol confirming activation of 5-HT_2C_ receptors. mCPP decreased intake of cookies eaten in the absence of hunger but had no effect on the amount of a pasta lunch consumed, although the pasta eating rate was reduced. mCPP also decreased BOLD responses to the sight of food pictures in reward-associated circuitry. These data suggest a role for 5-HT_2C_ receptor activation in mediating reward–related responses to food in humans.

Consistent with the present findings, Thomas et al., (2014) reported that mCPP did not reduce pasta intake but reduced rated appetite. By contrast, mCPP reduced intake of cookies and blunted the rated pleasantness of cookies. The effect of mCPP on microstructural measures of eating was also greater for cookie than for pasta consumption. 30mg mCPP reduced the rate of pasta consumption by 26% and cookie consumption by 39%, and increased the duration of pauses between mouthfuls of pasta and cookies by 49% and 109%, respectively. Taken together, these data suggest that the effect of mCPP on appetite was greater for the cookies than for the pasta.

mCPP increased ratings of nausea-like symptoms but these ratings were not correlated with food intake, suggesting they were unlikely to account for the effects on eating behaviour, as shown previously (Walsh et al., 1994; Thomas et a. 2014). In addition, the differential effects of mCPP on pasta and cookie intake suggests that these effects are not secondary to nausea which would be expected to suppress intake of both foods to a similar extent.

It is not possible to conclude whether the effects of mCPP were related to the greater palatability of the cookies, their greater energy density or both. Indeed, there are several differences between the pasta and cookies, including their sensory characteristics that might explain the pattern of results. In addition, the cookies were served after a satiating meal, and it may be that the effects of mCPP are enhanced under conditions of satiety. 5-HT_2C_ receptors may mediate reductions in food reward that occur as food is consumed, known as alliesthesia (Cabanac 1971). It will be important to test this hypothesis because reductions in reward-related responding that are specific to the satiated state are likely to be effective in helping individuals to curb their appetite but are unlikely to reduce hedonic responding in general as observed for the withdrawn anti-obesity drug rimonabant (Butler and Korbonits, 2009). Such investigations are also likely to shed light on the role of background neural activity and experimental context in the effects of 5-HT_2C_ receptor stimulation on reward-related behaviour (Vollm et al., 2010).

mCPP attenuated BOLD activity to the sight of both high and low food images in a number of brain regions involved in reward including the insula, anterior cingulate cortex, dorsolateral prefrontal cortex and caudate (Wang et al., 2008; Holroyd and Yeung, 2012; Morris et al., 2014). This pattern of results was not affected by adding nausea-like ratings as a co-variate in the analysis suggesting that the results are not explained by negative side effects of the drug. 5-HT_2C_ receptors are located in the prefrontal cortex, cingulate cortex and the caudate (Pazos, et al., 1987; Pompeiano et al., 1994; Marazziti et al., 1999), suggesting that mCPP may act in these areas to affect responding to food-stimuli. Areas showing increased activation after mCPP versus placebo were limited to the vmPFC, hippocampus, parahippocampal gyrus and amygdala. It has been proposed that activity in the vmPFC and dlPFC is associated with context-dependent value-based decision making and selective attention to motivationally relevant stimuli and the pattern of BOLD activity observed could suggest that the influence of contextual factors, such as metabolic state, on food valuation is altered by mCPP (Rudorf & Hare 2014; Walton et al. 2015). Since we found a stronger anorectic response after mCPP when eating in the absence of hunger, it would be of interest to examine whether the effect of mCPP on BOLD responses is dependent upon levels of satiety.

There was no effect of mCPP on hypothalamic responses but we draw no strong conclusion about this null effect because the hypothalamus is difficult to image and is susceptible to artifacts due to its proximity to the sinuses (Ojemann, et al. 1997), which might also explain the lack of a main effect of task for hypothalamic responding. A recent study of the effects of lorcaserin on BOLD responses to food pictures in participants with obesity similarly failed to find an effect on hypothalamic activation (Farr et al. 2016). However, participants who received lorcaserin twice a day showed less activation in insula, parietal cortex, visual cortices, hippocampus and amygdala in the fasting state at 1 week than 4 weeks. Although the study of Farr and colleagues (2016) is not directly comparable to the present study, due to several methodological differences between the studies, the data suggest that there is some overlap between the effects of mCPP and lorcaserin on responding to food pictures in the short term, which could point towards common 5-HT_2C_ receptor-mediated effects.

An exploratory post-hoc analysis examined apparent individual variability in response to mCPP. At baseline, participants who did not respond to mCPP by decreasing their intake of cookies showed greater BOLD activity than responders in areas including dorsolateral prefrontal cortex, insula and putamen and midbrain. After dosing with mCPP, non-responders rated cookies as more pleasant than responders, in the absence of differences in rated hunger suggesting that heightened reward response might be responsible for blunting the hypophagic effect of mCPP. Further investigation of the characteristics of responders versus non-responders is required, but our results suggest that imaging data may shed light on which individuals are likely to show reduced food intake after treatment with 5-HT_2C_ receptor agonists.

We provide the first evidence that mCPP reduces consumption of a palatable energy dense snack in humans. mCPP also caused a marked reduction in neural activity across reward-related brain regions to the sight of food. An implication of these findings is that 5-HT_2C_ receptor agonists such as lorcaserin, may be effective in helping individuals to reduce their intake of palatable food in the absence of hunger. In addition, we found that some participants did not reduce their cookie intake after treatment with mCPP and this was associated with enhanced rated cookie pleasantness, and enhanced baseline BOLD responses to food in key reward areas. Further investigation of stratification of responding to mCPP (and potentially other 5-HT_2C_ receptor agonists such as lorcaserin) is required to identify patients who are more likely to respond to weight management drugs that act at the 5-HT_2C_ receptor and hence more effectively target therapy.

Funding and Disclosure

Dr Colin Dourish is an employee, Director and shareholder of P1vital Ltd., Dr Suzanne Higgs is a member of P1vital's Advisory Panel and Dr Jason Michael Thomas was funded by the Steve Cooper P1vital—BBSRC PhD Studentship. No other conflicts are reported. This work was supported by P1vital, the Biotechnology and Biological Sciences Research Council (BBSRC) and the University of Birmingham. Dr Thomas is now based at Aston University.

Acknowledgements

The research was carried out at the National Institute for Health Research (NIHR)/Wellcome Trust Birmingham Clinical Research Facility. The views expressed are those of the authors and not necessarily those of the NHS, the NIHR or the Department of Health. The authors would like to thank the staff at the facility for their support during the study.

**References**

Allen HA, Chambers A, Blissett J, Chechlacz M, Barrett T, Higgs S, Nouwen, A (2016) Relationship between Parental Feeding Practices and Neural Responses to Food Cues in Adolescents. *PLoS ONE* 11: e0157037.

Aronson SC, Black JE, McDougle CJ, Scanley BE, Heninger GR, Price LH, et al. (1995). Serotonergic mechanisms of cocaine effects in humans. *Psychopharmacology,* 119(2), 179-185.

Batterham RL, Ffytche, DH, Rosenthal, JM, Zelaya, FO, Barker, GJ, Withers, DJ Williams, SCR (2007) PYY modulation of cortical and hypothalamic brain areas predicts feeding behaviour in humans. *Nature* 450: 106-109.

Beck AT, Ward CH, Mendelson M, Mock J, Erbaugh J (1961) An inventory for measuring depression. *Arch. Gen. Psychiat.* 4: 561–571.

Berthoud, HR (2011) Metabolic and hedonic drives in the neural control of appetite: who is the boss? *Curr. Opin. Neurobiol.* 21: 888-896.

Bubar MJ, Cunningham, KA (2007) Distribution of serotonin 5-HT_2C_ receptors in the ventral tegmental area. *Neuroscience* 146: 286-297.

Butler H, Korbonits M (2009) Cannabinoids for clinicians: the rise and fall of the cannabinoid antagonists. *Eur. J. Endocrinol.* 161: 655-62.

Cabanac M (1971) Physiological role of pleasure. *Science,* 173: 1103-1107.

Cowen PJ, Sargent PA, Williams C, Goodall EM, Orlikov AB (1995) Hypophagic, endocrine and subjective responses to m-chlorophenylpiperazine in healthy men and women. *Hum. Psychopharm*. 10: 385-391.

Cox RW (1996) AFNI: Software for analysis and visualization of functional magnetic resonance neuroimages. *Comput. Biomed. Res.* 29: 162–73.

Eysenck HJ, Eysenck SBG (1975) *Manual of the Eysenck Personality Questionnaire (adult and junior).* London: Hodder & Stoughton.

Farooqi IS, Bullmore E, Keogh J, Gillard J, O'Rahilly S, Fletcher PC (2007) Leptin regulates striatal regions and human eating behavior. *Science* 317: 1355.

Farr, O. M., Upadhyay, J., Gavrieli, A., Camp, M., Spyrou, N., Kaye, H., ... & Srnka, A. (2016). Lorcaserin administration decreases activation of brain centers in response to food cues and these emotion-and salience-related changes correlate with weight loss effects: a 4-week-long randomized, placebo-controlled, double-blind clinical trial. Diabetes, 65(10), 2943-2953.

Fletcher PJ, Chintoh AF, Sinyard J, Higgins GA (2004) Injection of the 5-HT2C receptor agonist Ro60-0175 into the ventral tegmental area reduces cocaine-induced locomotor activity and cocaine self-administration. *Neuropsychopharmacology* 29: 308-318

Goldstone AP, de Hernandez CG, Beaver JD, Muhammed K, Croese C, Bell G, Durighel G, Hughes E, Waldman AD, Frost G, Bell JD (2009) Fasting biases brain reward systems towards high-calorie foods. *Eur. J. Neurosci.* 30: 1625–1635.

Guthoff M, Grichisch Y, Canova C, Tschritter O, Veit R, Hallschmid M, Häring HU, Preissl H, Hennige AM, Fritsche A (2010) Insulin modulates food-related activity in the central nervous system. *J. Clin. Endocrinol. Metab.* 95: 748-55.

Heisler, L. K., Jobst, E. E., Sutton, G. M., Zhou, L., Borok, E., Thornton-Jones, Z., ... & Lee, C. E. (2006). Serotonin reciprocally regulates melanocortin neurons to modulate food intake. *Neuron*, 51(2), 239-249.

Heisler LK, Cowley MA, Tecott LH, Fan W, Low MJ, Smart JL, Rubinstein M, Tatro JB, Marcus JN, Holstege H, Lee CE, Cone RD, Elmquist JK (2002) Activation of Central Melanocortin Pathways by Fenfluramine*. Science* 297: 609–611.

Higgins GA, Silenieks LB, Lau W, de Lannoy IAM, Lee DKH, Izhakova J, Fletcher, PJ (2013) Evaluation of chemically diverse 5-HT2C receptor agonists on behaviours motivated by food and nicotine and on side effect profiles. *Psychopharmacology* 226: 475-490.

Holroyd CB, Yeung N (2012) Motivation of extended behaviors by anterior cingulate cortex. *Trends Cogn. Sci.* 16: 122-8.

Lowe MR, Butryn ML, Didie ER, Annunziato RA, Thomas JG, Crerand CE, Ochner CN, Coletta MC, Bellace D, Wallaert M, Halford J (2009) The Power of Food Scale. A new measure of the psychological influence of the food environment. *Appetite* 53: 114-118.

Maldjian JA, Laurienti PJ, Kraft RA, Burdette JH (2003) An automated method for neuroanatomic and cytoarchitectonic atlas-based interrogation of fmri data sets. *NeuroImage* 19: 1233– 1239.

Malik S, McGlone F, Bedrossian D, Dagher A (2008) Ghrelin modulates brain activity in areas that control appetitive behavior. *Cell. Metab.* 7: 400-9.

Marazziti D, Rossi A, Giannaccini G, Zavaglia KM, Dell'Osso L, Lucacchini A, Cassano GB (1999) Distribution and characterization of [3H]mesulergine binding in human brain postmortem. *Eur. Neuropsychopharmacol.* 10: 21–26.

Meltzer, H. Y., & Maes, M. (1995). Pindolol pretreatment blocks stimulation by meta-chlorophenylpiperazine of prolactin but not cortisol secretion in normal men. Psychiatry research, 58(2), 89-98.

Morris RW, Dezfouli A, Griffiths KR, Balleine BW (2014) Action-value comparisons in the dorsolateral prefrontal cortex control choice between goal-directed actions. *Nat. Commun*. 5: 4390.

Nelson HE (1982) National Adult Reading Test (NART): *For the Assessment of Premorbid Intelligence in Patients with Dementia: Test Manual.* Windsor, UK: NFER-Nelson.

Ojemann, J. G., Akbudak, E., Snyder, A. Z., McKinstry, R. C., Raichle, M. E., & Conturo, T. E. (1997). Anatomic localization and quantitative analysis of gradient refocused echo-planar fMRI susceptibility artifacts. Neuroimage, 6(3), 156-167.

Patton JH, Stanford MS, Barratt ES (1995) Factor structure of the Barratt impulsiveness scale. *J. Clin. Psychol.* 51: 768–774.

Pompeiano M, Palacios JM, Mengod G (1994) Distribution of the serotonin 5-HT2

receptor family mRNAs: comparison between 5-HT2A and 5-HT2C receptors. *Brain. Res.*

*Mol. Brain. Res.* 23: 163-78.

Pazos A, Probst A, Palacios, JM (1987) Serotonin receptors in the human brain--IV. Autoradiographic mapping of serotonin-2 receptors. *Neuroscience* 21: 123-39.

Rudorf, S., & Hare, T. A. (2014). Interactions between Dorsolateral and Ventromedial Prefrontal Cortex Underlie Context-Dependent Stimulus Valuation in Goal-Directed Choice. *The Journal of Neuroscience,* 34(48), 15988-15996.

Sohn JW, Xu Y, Jones JE, Wickman K, Williams KW, Elmquist JK (2011) Serotonin 2C receptor activates a distinct population of arcuate pro-opiomelanocortin neurons via TRPC channels. *Neuron* 71: 488-97.

Spitzer RL, Williams JB, Gibbon M, First MB (2004) *Structured clinical interview for the DSM-IV (SCID-I/P).* New York: Biometrics Research, New York State Psychiatric Institute.

Spielberger CD (1983) *Manual for the State-Trait Anxiety Inventory.* Palo Alto, CA: Consulting Psychologists Publishing.

Stunkard AJ, Messick S (1985) The three-factor eating questionnaire to measure dietary restraint disinhibition and hunger. *J. Psychosom. Res.* 29: 71-83.

Thomas JM, Higgs S, Dourish CT, Hansen P, Harmer CJ, McCabe C (2015) Satiation attenuates BOLD activity in brain regions involved in reward and increases activity in an inhibitory control area: an fMRI study in healthy volunteers. *Am. J. Clin. Nutr.* 101: 697-704.

Thomas JM, Dourish CT, Tomlinson J, Hassan-Smith Z, Higgs S (2014) Effects of the 5-HT_2C_ receptor agonist meta-chlorophenylpiperazine on appetite, food intake and emotional processing in healthy volunteers. *Psychopharmacology (Berl)* 231: 2449-59.

van der Laan LN, de Ridder DT, Viergever MA, Smeets PA (2011). The first taste is always with the eyes: a meta-analysis on the neural correlates of processing visual food cues. *Neuroimage,* 55: 296-303.

Vollm B, Richardson P, McKie S, Reniers R, Elliott R, Anderson IM, Williams S, Dolan M, Deakin B (2010) Neuronal correlates and serotonergic modulation of behavioural inhibition and reward in healthy and antisocial individuals. *J. Psychiatr. Res.* 23: 123–131.

von Zerssen D, Strian F, Schwarz D (1974) Evaluation of depressive states, especially in longitudinal studies. *Mod. Probl. Pharmacopsychiatry* 7: 189–202.

Walsh AE, Smith KA, Oldman AD, Williams C, Goodall EM, Cowen PJ (1994) m-Chlorophenylpiperazine decreases food intake in a test meal. *Psychopharmacology* 116: 120-122.

Walton ME, Chau BK, Kennerley SW (2015) Prioritising the relevant information for learning and decision making within orbital and ventromedial prefrontal cortex. *Curr. Opin. Behav.* Sci. 1: 78-85.

Wang GJ, Tomasi D, Backus W, Wang R, Telang F, Geliebter A, Korner J, Bauman A, Fowler JS, Thanos PK & Volkow ND (2008) Gastric distention activates satiety circuitry in the human brain*. NeuroImage* 39: 1824–1831.

Watson D, Clark LA, Tellegen A (1988) Development and validation of brief measures of positive and negative affect: The PANAS scales. *J. Pers. Soc. Psychol.* 54: 1063-1070.

**Legends**

**Figure 1** Flow-diagram for screening process followed by an overview of key events and timings for test days in hours (hrs).

**Figure 2** Universal Eating Monitor measures for the pasta lunch (Panel A) and cookie snack (Panel B) split by placebo and mCPP conditions. mCPP significantly reduced the consumption of cookies but not pasta. For both foods, mCPP significantly increased the pause between mouthfuls and reduced the eating rate (amount eaten per minute). Time spent eating was not significantly different for either food after dosing with mCPP. * *p* < 0.05** *p* < 0.01; *** *p* < 0.001

**Figure 3** BOLD response to high and low calorie food images (Panel A and Panel B, respectively). Orange depicts brain areas where the BOLD response was greater after dosing with mCPP compared to placebo whereas blue depicts brain areas where participants show a greater BOLD response when dosed with placebo compared to mCPP; (L = left; R = right; dlPFC = dorsolateral prefrontal cortex; IFG = inferior frontal gyrus; MFG = middle frontal gyrus; ACC = anterior cingulate cortex; IFG = inferior frontal gyrus; vmPFC = ventromedial prefrontal cortex).

**Figure 4** Baseline BOLD response to high calorie food images. Orange depicts brain areas where non-responders show a greater BOLD response than responders; Blue depicts brain areas where responders show a greater BOLD response than non-responders (L = left; R = right; VTA = ventral tegmental area; dlPFC = dorsolateral prefrontal cortex; MFG = middle frontal gyrus; IFG = inferior frontal gyrus; vmPFC = ventromedial prefrontal cortex).

**Illustrations and Tables**


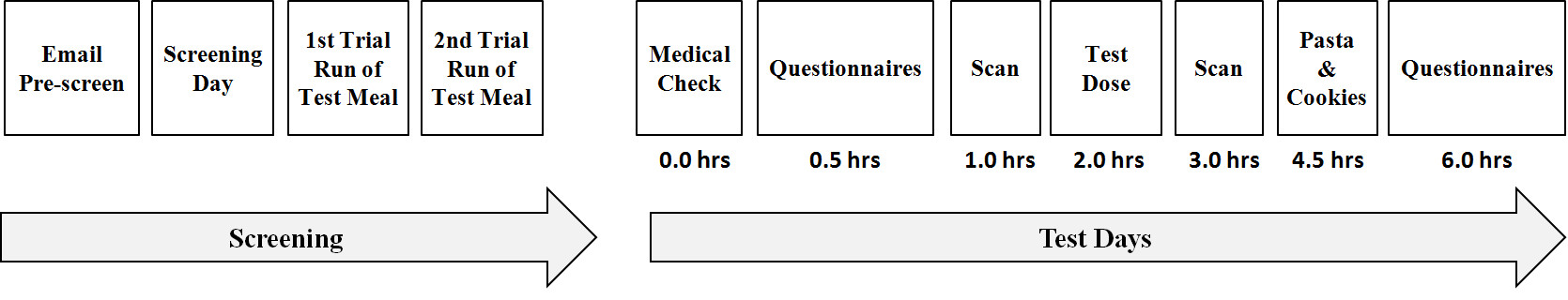


**Figure 1**

**
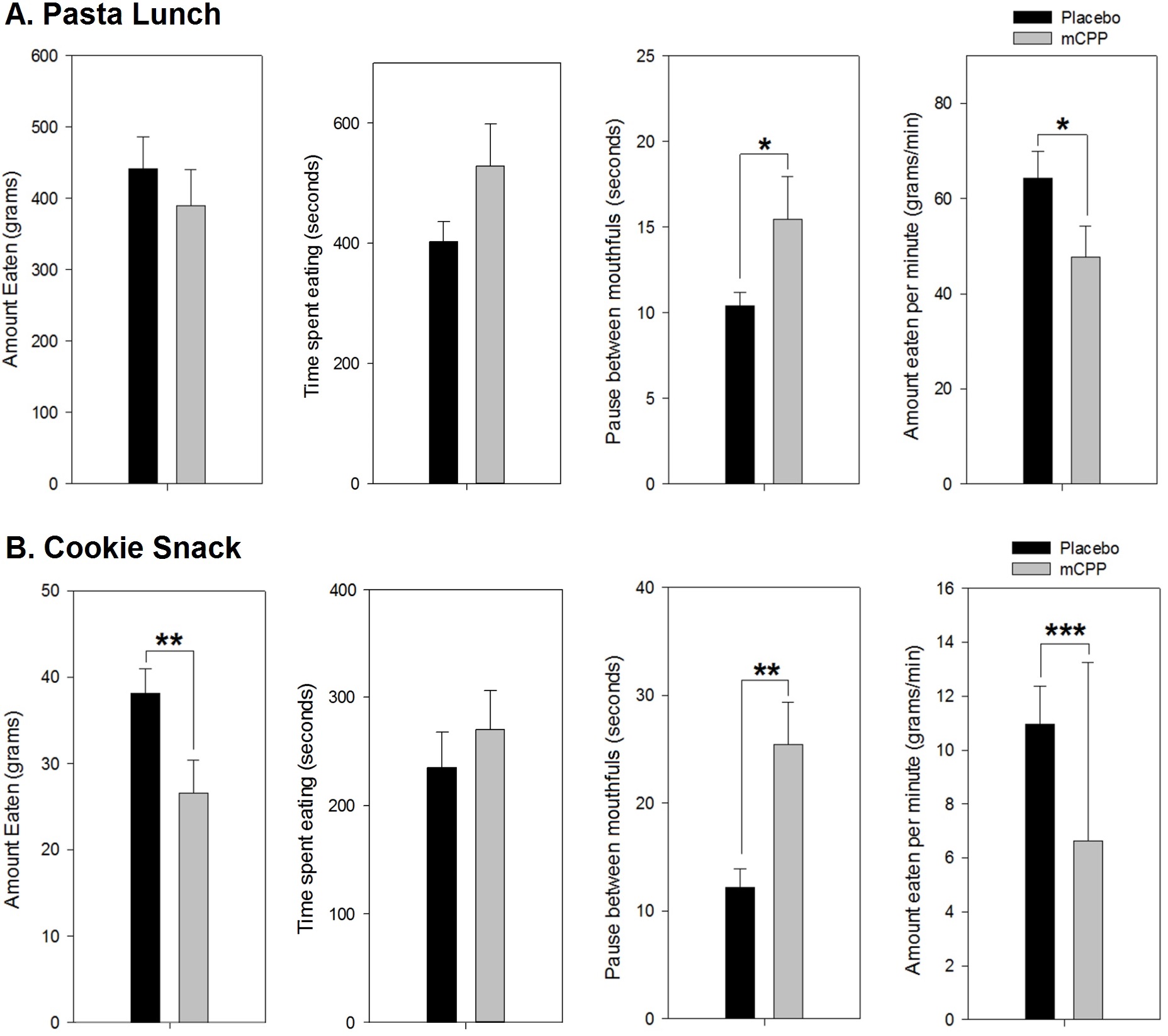
**

**Figure 2**

**
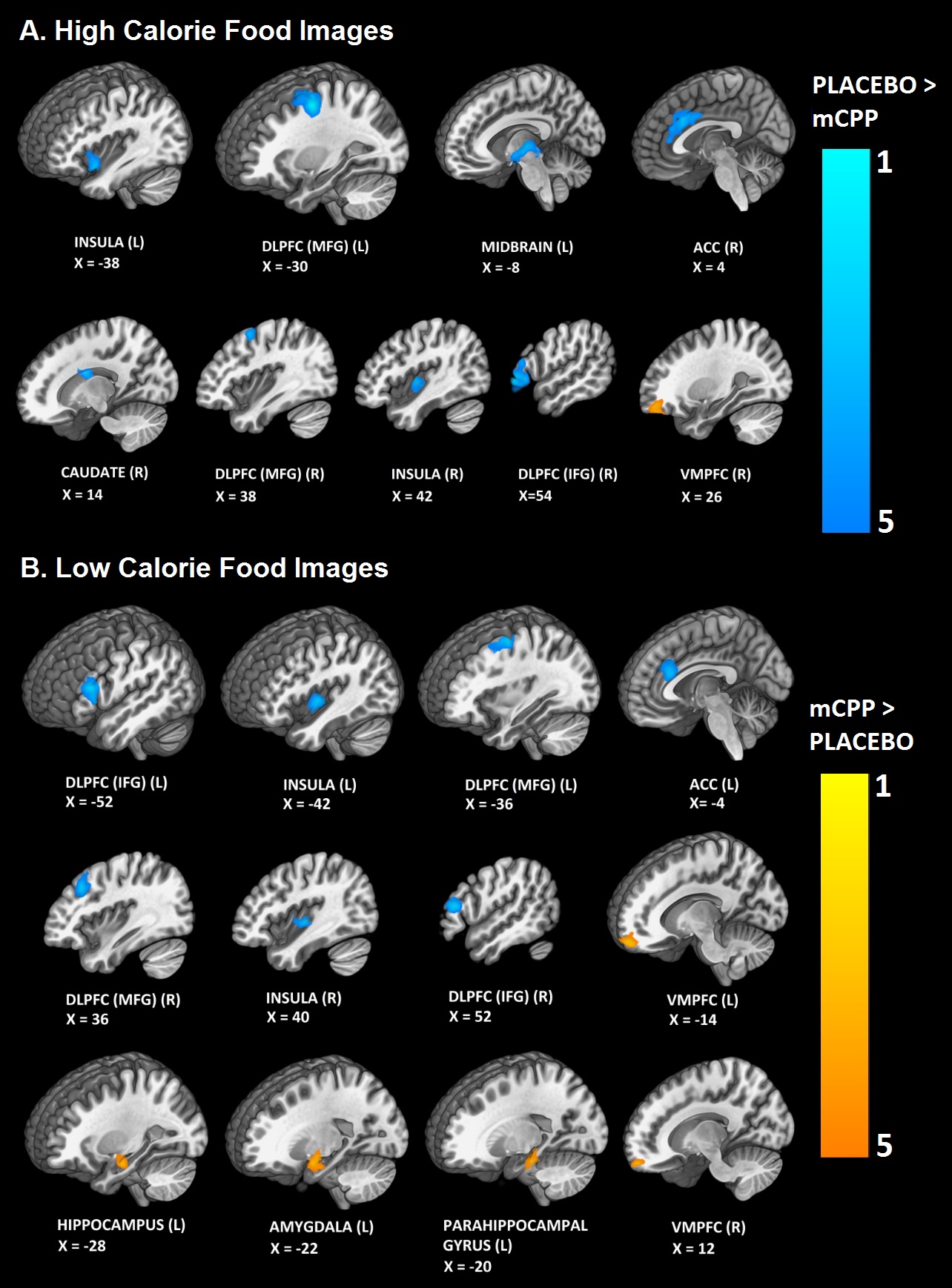
**

**Figure 3**


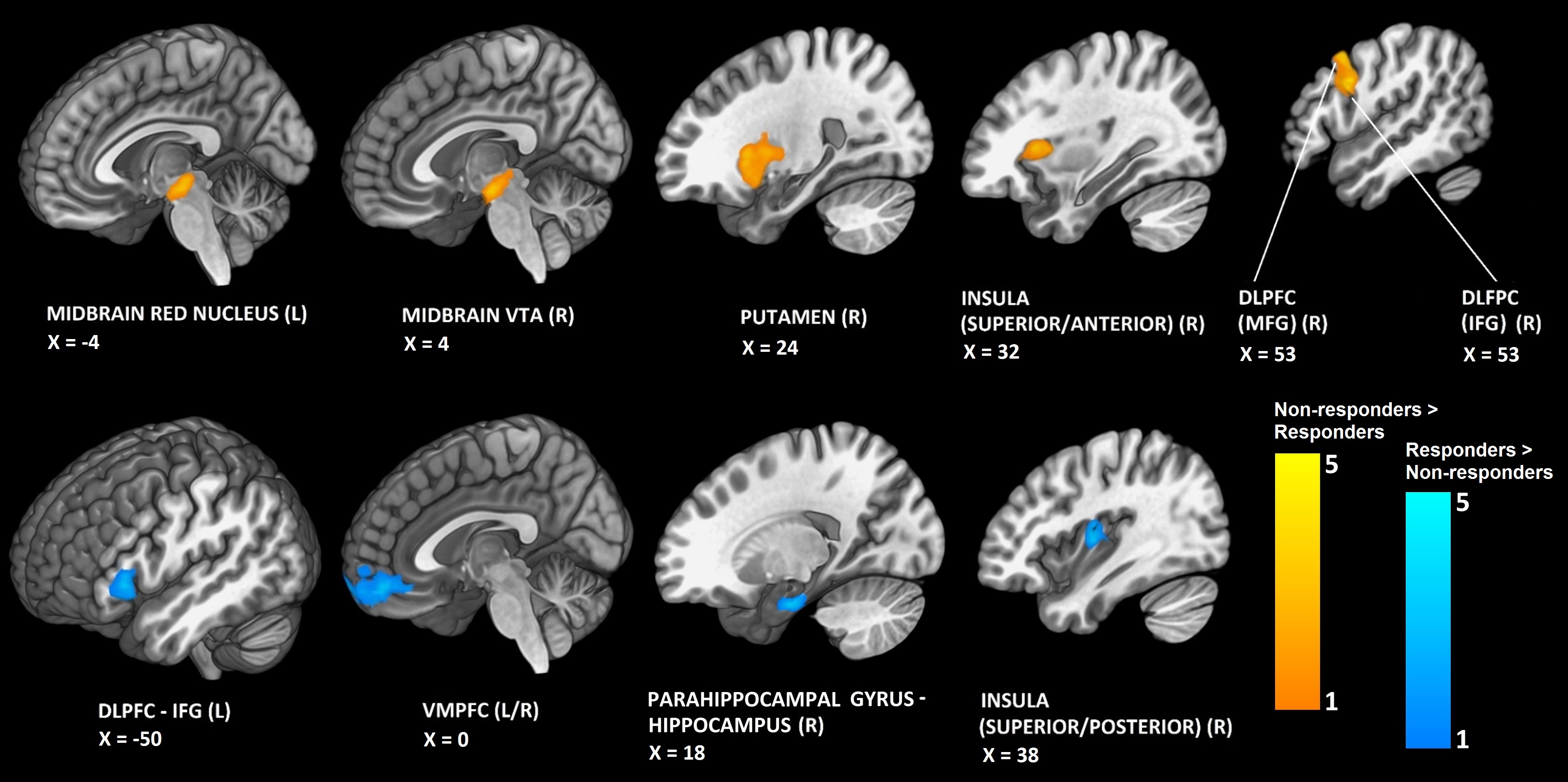


**Figure 4**

| **Table 1** Local maxima of key appetitive & reward areas showing: (A) main effect of condition (placebo versus mCPP), split by activity to high and low calorie food images; (B) differences in BOLD signal between non-responders and responders at baseline to the sight of high calorie food images. | | | | | | | | |
| --- | --- | --- | --- | --- | --- | --- | --- | --- |
|  |  |  |  |  |  |  |  |  |
| **Brain Region (Hemisphere)** | | **Montreal Neurological Institute (MNI) Coordinates** | | | **Brodmann Area** | | | **Z-Score** |
|  |  | **X** | **Y** | **Z** | |  |  | |
|  | |  |  |  |  | | |  |
| **A. MAIN EFFECT OF CONDITION** | |  |  |  |  | | |  |
| **Reduced activation after mCPP (compared to placebo)** | | | | | | | | |
| ***High Calorie Food Images*** | |  | | |  | | |  |
| dlPFC (Middle Frontal Gyrus) / Precentral Gyrus (L) | | -30 | -6 | 54 | 6 | | | 5.2 |
| Anterior Cingulate Cortex (R) | | 4 | 22 | 30 | 24 | | | 3.3 |
| Insula (L) | | -38 | 10 | -14 | 48 | | | 3.3 |
| Caudate (R) | | 14 | -2 | 24 | -- | | | 3.1 |
| dlPFC (Middle Frontal Gyrus) (R) | | 38 | 6 | 58 | 6 | | | 3.0 |
| dlPFC (Inferior Frontal Gyrus) (R) | | 54 | 28 | 0 | 45 | | | 2.8 |
| Insula (R) | | 42 | -10 | 0 | 48 | | | 2.8 |
| Midbrain (L) | | -8 | -14 | -4 | -- | | | 2.7 |
| ***Low Calorie Food Images*** | |  | | |  | | |  |
| dlPFC (Inferior Frontal Gyrus) (L) | | -52 | 26 | 20 | 45 | | | 3.9 |
| dlPFC (Inferior Frontal Gyrus) (R) | | 52 | 32 | 16 | 48 | | | 3.9 |
| dlPFC (Middle Frontal Gyrus) (L) | | -36 | -2 | 62 | 6 | | | 3.6 |
| dlPFC (Middle Frontal Gyrus) (R) | | 36 | 26 | 42 | 9 | | | 3.5 |
| Insula (L) | | -42 | -8 | 2 | 48 | | | 3.4 |
| Insula (R) | | 40 | -14 | 0 | 48 | | | 3.3 |
| Anterior Cingulate Cortex (L) | | -4 | 26 | 28 | 24 | | | 3.1 |
|  | |  |  |  |  | | |  |
| **Increased activation after mCPP (compared to placebo)** | | | | | | | | |
| ***High Calorie*** |  |  | | |  | | |  |
| Ventromedial Prefrontal Cortex (R) | | 26 | 52 | -12 | 11 | | | 3.1 |
| ***Low Calorie*** |  |  | | |  | | |  |
| vmPFC (L) | | -14 | 62 | -16 | 11 | | | 3.3 |
| Parahippocampal Gyrus (L) | | -20 | -26 | -22 | 30 | | | 3.2 |
| Hippocampus (L) | | -28 | -14 | -20 | 20 | | | 3.2 |
| Amygdala (L) | | -22 | -6 | -18 | 34 | | | 3.1 |
| vmPFC (R) | | 12 | 60 | -18 | 11 | | | 2.8 |
|  | |  |  |  |  | | |  |
| **B. RESPONDERS VERSUS NON-RESPONDERS** | | | | | | | | |
| **Non-Responders > Responders** | |  | | |  | | |  |
| Dorsolateral Prefrontal Cortex (Inferior Frontal Gyrus) (R) | | 52 | 12 | 30 | 44 | | | 4.4 |
| Dorsolateral Prefrontal Cortex (Middle Frontal Gyrus) (R) | | 54 | 14 | 44 | 44 | | | 4.0 |
| Brainstem (midbrain - ventral tegmental area) (R) | | 4 | -16 | -14 | -- | | | 3.6 |
| Brainstem (midbrain - red nucleus) (L) | | -4 | -20 | -12 | -- | | | 3.5 |
| Insula (superior/anterior) (R) | | 32 | 22 | 8 | 48 | | | 3.4 |
| Putamen (R) | | 24 | 14 | 0 | 48 | | | 3.1 |
| **Responders > Non-Responders** | |  | | |  | | |  |
| Ventromedial prefrontal cortex | | 0 | 54 | -12 | 11 | | | 3.7 |
| Insula (superior/posterior) (R) | | 38 | -8 | 6 | 48 | | | 3.6 |
| Dorsolateral Prefrontal Cortex (Inferior Frontal Gyrus) (L) | | -50 | 26 | 2 | 45 | | | 3.6 |
| Parahippocampal gyrus / Hippocampus (R) | | 18 | -10 | -26 | 28 | | | 3.5 |
|  | |  |  |  |  | | |  |
| FWE cluster corrected (voxel *p* < 0.005; cluster > 24 contiguous voxels – *p* < 0.05). Left side (L); Right side (R); | | | | | | | | |

**Supplemental Information**

| **Supplemental Table 1:** Main effect of task for each region of interest and p values indicating statistical significance (mean and standard error of the mean) | | | | | |
| --- | --- | --- | --- | --- | --- |
|  |  |  |  |  |  |
|  |  |  |  |  |  |
| Region of Interest | Food Images | | Control Images | | Bonferroni corrected *p* value |
|  | Mean | SEM | Mean | SEM |  |
|  |  |  |  |  |  |
| Nucleus Accumbens | -0.07 | 0.02 | -0.15 | 0.03 | *p* < 0.01 |
| Midbrain | -0.06 | 0.02 | -0.11 | 0.01 | *p* < 0.05 |
| Orbitofrontal Cortex | 0.04 | 0.02 | -0.01 | 0.02 | *p* < 0.05 |
| Ventromedial Prefrontal Cortex | 0.04 | 0.04 | -0.02 | 0.04 | *p* < 0.05 |
| Insula | -0.02 | 0.02 | -0.09 | 0.02 | *p* < 0.01 |
| Hypothalamus | -0.03 | 0.02 | -0.05 | 0.01 | *p* > 0.05 |
| Amygdala | 0.13 | 0.03 | 0.04 | 0.02 | *p* < 0.05 |
| Cingulate Cortex | -0.04 | 0.02 | -0.12 | 0.02 | *p* < 0.01 |
| Hippocampus / Parahippocampal Gyrus | 0.07 | 0.01 | 0.03 | 0.02 | *p* > 0.05 |
| Dorsal Striatum | -0.04 | 0.02 | -0.09 | 0.02 | *p* < 0.05 |
| Dorsolateral Prefrontal Cortex | 0.01 | 0.02 | -0.06 | 0.02 | *p* < 0.01 |
|  |  |  |  |  |  |
| Note 1: Region of interest (ROI) masks selected from standard templates from WFUPickatlas  (Maldjian et al., 2003).  Note 2: ROIs based on previous literature: Fletcher et al. 2010; Thomas et al. 2015; Haase et al. 2009 LaBar et al., 2001; Goldstone et al., 2009; Führer et al., 2008 Kringelbach et al., 2003; Killgore et al., 2003; Del Parigi et al., 2005; Gautier et al., 2000; Cornier et al., 2009; Simmons et al., 2005; Porubska et al., 2006. | | | | | |

**References for Supplemental Table 1**

Cornier MA, Salzberg AK, Endly DC, Bessesen DH, Rojas DC, Tregellas JR (2009) The effects of overfeeding on the neuronal response to visual food cues in thin and reduced-obese individuals. PLoS One 4: e6310.

Del Parigi A, Chen K, Salbe AD, Reiman EM, Tataranni PA (2005) Sensory experience of food and obesity: a positron emission tomography study of the brain regions affected by tasting a liquid meal after a prolonged fast. Neuroimage 15: 436-43.

Fletcher PC, Napolitano A, Skeggs A, Miller SR, Delafont B, Cambridge VC, de Wit S, Nathan PJ, Brooke A, O'Rahilly S, Farooqi IS, Bullmore, ET (2010) Distinct modulatory effects of satiety and sibutramine on brain responses to food images in humans: a double dissociation across hypothalamus, amygdala, and ventral striatum. J. Neurosci. 30: 14346-55.

Führer D, Zysset S, Stumvoll M (2008) Brain Activity in Hunger and Satiety: An Exploratory Visually Stimulated fMRI Study. Obesity 16: 945-950.

Gautier JF, Chen K, Salbe AD, Bandy D, Pratley RE, Heiman M, Ravussin E, Reiman EM, Tataranni PA (2000) Differential brain responses to satiation in obese and lean men. Diabetes 49: 838-46.

Haase L, Cerf-Ducastel B, Murphy C (2009) Cortical activation in response to pure taste stimuli during the physiological states of hunger and satiety. Neuroimage 44: 1008-1021.

Killgore WD, Young AD, Femia LA, Bogorodzki P, Rogowska J, Yurgelun-Todd DA (2003) Cortical and limbic activation during viewing of high- versus low calorie foods. Neuroimage 19: 1381–1394.

Kringelbach ML, O’Doherty J, Rolls ET, Andrews C (2003) Activation of the human orbitofrontal cortex to a liquid food stimulus is correlated with its subjective pleasantness. Cereb. Cortex 13: 1064–1071.

LaBar KS, Gitelman DR, Parrish TB, Kim YH, Nobre AC, Mesulam MM (2001) Hunger selectively modulates corticolimbic activation to food stimuli in humans. Behav. Neurosci. 115: 493–500.

Maldjian JA, Laurienti PJ, Kraft RA, Burdette JH (2003) An automated method for neuroanatomic and cytoarchitectonic atlas-based interrogation of fmri data sets. NeuroImage 19: 1233– 1239.

Porubska K, Veit R, Preissl H, Fritsche A, Birbaumer N (2006) Subjective feeling of appetite modulates brain activity: an fMRI study. Neuroimage 32: 1273–1280.

Simmons WK, Martin A, Barsalou LW (2005) Pictures of appetizing foods activate gustatory cortices for taste and reward. Cereb. Cortex 15: 1602– 1608.

Thomas JM, Higgs S, Dourish CT, Hansen P, Harmer CJ, McCabe C (2015) Satiation attenuates BOLD activity in brain regions involved in reward and increases activity in an inhibitory control area: an fMRI study in healthy volunteers. Am. J. Clin. Nutr. 101: 697-704.

| **Supplemental Table 2** Questionnaire scores (averaged across test days pre and post dosing) | |
| --- | --- |
| Measure | Value |
| BDI |  |
| Mean (SEM) | 1.9 (0.75) |
| Range | 0 - 11.5 |
|  |  |
| BIS-11 |  |
| Mean (SEM) | 59.2 (2.0) |
| Range | 39.8 - 83.0 |
|  |  |
| BFS |  |
| Mean (SEM) | 14.1 (2.6) |
| Range | 0.3 - 51.0 |
|  |  |
| PFS |  |
| Mean (SEM) | 33.7 (2.4) |
| Range | 20.5 - 63.3 |
|  |  |
| PANAS-Positive |  |
| Mean (SEM) | 30.4 (1.5) |
| Range | 17.3 - 46.3 |
|  |  |
| PANAS-Negative |  |
| Mean (SEM) | 11.8 (0.8) |
| Range | 10.0 - 28.0 |
|  |  |
| STAI-State |  |
| Mean (SEM) | 26.8 (0.9) |
| Range | 20.8 - 41.8 |
|  |  |
| STAI-Trait |  |
| Mean (SEM) | 30.7 (1.7) |
| Range | 20.8 - 57.0 |
|  |  |
|  |  |
| BDI - Beck Depression Inventory; BIS 11 - Barratt Impulsiveness Scale; BFS- Befindlichskeit scale of mood and energy; PFS - Power of Food Scale; PANAS - Positive and Negative Affect Schedule; STAI - State Trait Anxiety Inventory | |
|  |  |
|  |  |

| **Supplemental Table 3** Area Under The Curve (AUC) scores for VAS items, split by mCPP and Placebo conditions (standard error of the mean) | | |
| --- | --- | --- |
| VAS Item | Placebo | mCPP |
| Alertness | 14520.22 (892.65) | 13252.39 (912.46) |
| Disgust | 601.52 (74.80) | 912.17 (198.66) |
| Drowsiness | 3346.52 (672.08) | 3450.87 (606.22) |
| Nausea | 606.09 (90.63) | 2158.04 (317.22)*** |
| Faint | 706.30 (105.49) | 2254.13 (582.79)** |
| Lightheaded | 751.09 (121.85) | 2938.26 (514.16)*** |
| Anxiety | 603.04 (82.50) | 717.61 (141.54) |
| Happiness | 15632.17 (447.76) | 14662.39 (712.02) |
| Sadness | 740.22 (128.02) | 731.74 (128.88) |
| Withdrawn | 787.83 (124.36) | 787.17 (170.62) |
| Hunger | 8172.39 (502.31) | 6596.30 (718.66)* |
| Desire to Eat | 7830.65 (599.53) | 6168.04 (726.58)* |
| Fullness | 10401.74 (650.88) | 10176.96 (829.69) |
| Thirst | 4685.46 (582.40) | 4594.13 (635.99) |
|  |  |  |

**p* < 0.05; ***p* < 0.01; *** *p* < 0.001

| **Supplemental Table 4** Characteristics and ratings from participants split by responders and non-responders (standard error of the mean) | | |
| --- | --- | --- |
| Measure | Responders | Non-responders |
|  |  |  |
| Age | 24.3 (2.1) | 20.9 (1.2) |
| Body Mass Index (BMI) | 21.6 (0.4) | 22.1 (0.6) |
| TFEQ Cognitive Restraint | 5.5 (0.8) | 5.8 (0.8) |
| TFEQ Disinhibition | 6.6 (1.3) | 8.1 (1.0) |
| TFEQ Hunger | 5.5 (1.3) | 7.6 (1.1) |
| BDI | 0.9 (0.5) | 1.6 (1.0) |
| BIS-11 | 57.6 (2.5) | 57.6 (2.1) |
| BFS | 9.8 (2.7) | 12.6 (6.2) |
| PFS | 30.1 (2.5) | 35.4 (3.4) |
| PANAS-Positive | 32.4 (2.4) | 27.7 (2.4) |
| PANAS-Negative | 10.6 (0.2) | 11.2 (0.6) |
| STAI-State | 25.5 (1.0) | 28.1 (2.2) |
| STAI-Trait | 28.0 (1.3) | 33.4 (3.0) |
|  |  |  |
| *Visual Analogue Scales* |  |  |
| Alertness | 72.5 (3.0) | 68.0 (9.6) |
| Disgust | 7.1 (3.3) | 1.8 (0.5) |
| Drowsiness | 12.4 (3.1) | 7.0 (3.3) |
| Nausea | 8.1 (3.6) | 1.6 (0.5) |
| Faint | 4.0 (0.6) | 3.6 (1.1) |
| Lightheaded | 4.2 (0.8) | 2.7 (0.8) |
| Anxiety | 6.4 (1.9) | 4.5 (1.4) |
| Happiness | 70.1 (4.0) | 74.8 (1.4) |
| Sadness | 5.1 (1.3) | 3.2 (1.4) |
| Withdrawn | 7.1 (2.2) | 2.8 (1.0) |
| Hunger | 17.8 (3.7) | 17.7 (4.8) |
| Desire to Eat | 16.0 (12.8) | 12.6 (9.2) |
| Fullness | 65.2 (7.8) | 75.7 (4.4) |
| Thirst | 33.4 (18.4) | 20.9 (15.7) |
|  |  |  |
| *Cookie Snack* |  |  |
| Hunger Rating | 21.6 (6.0) | 21.1 (6.6) |
| Pleasantness Rating | 70.9 (4.9) | 85.3 (3.4)* |
|  |  |  |
|  |  |  |
| TFEQ - Three Factor Eating Questionnaire; BDI - Beck Depression Inventory; BIS 11 - Barratt Impulsiveness Scale; BFS- Befindlichskeit scale of mood and energy; PFS - Power of Food Scale; PANAS - Positive and Negative Affective Schedule; STAI - State Trait Anxiety Inventory. * *p* < 0.05 | | |
|  |  |  |
